# HESML: a real-time semantic measures library for the biomedical domain with a reproducible survey

**DOI:** 10.1186/s12859-021-04539-0

**Published:** 2022-01-06

**Authors:** Juan J. Lastra-Díaz, Alicia Lara-Clares, Ana Garcia-Serrano

**Affiliations:** NLP & IR Research Group, E.T.S.I. Informática, Universidad Nacional de Educación a Distancia (UNED), C/Juan del Rosal 16, 28040 Madrid, Spain

**Keywords:** HESML, Semantic measures library, Ontology-based semantic similarity measures, Information content models, SNOMED-CT, MeSH, Gene ontology, WordNet

## Abstract

**Background:**

Ontology-based semantic similarity measures based on SNOMED-CT, MeSH, and Gene Ontology are being extensively used in many applications in biomedical text mining and genomics respectively, which has encouraged the development of semantic measures libraries based on the aforementioned ontologies. However, current state-of-the-art semantic measures libraries have some performance and scalability drawbacks derived from their ontology representations based on relational databases, or naive in-memory graph representations. Likewise, a recent reproducible survey on word similarity shows that one hybrid IC-based measure which integrates a shortest-path computation sets the state of the art in the family of ontology-based semantic measures. However, the lack of an efficient shortest-path algorithm for their real-time computation prevents both their practical use in any application and the use of any other path-based semantic similarity measure.

**Results:**

To bridge the two aforementioned gaps, this work introduces for the first time an updated version of the HESML Java software library especially designed for the biomedical domain, which implements the most efficient and scalable ontology representation reported in the literature, together with a new method for the approximation of the Dijkstra’s algorithm for taxonomies, called Ancestors-based Shortest-Path Length (AncSPL), which allows the real-time computation of any path-based semantic similarity measure.

**Conclusions:**

We introduce a set of reproducible benchmarks showing that HESML outperforms by several orders of magnitude the current state-of-the-art libraries in the three aforementioned biomedical ontologies, as well as the real-time performance and approximation quality of the new AncSPL shortest-path algorithm. Likewise, we show that AncSPL linearly scales regarding the dimension of the common ancestor subgraph regardless of the ontology size. Path-based measures based on the new AncSPL algorithm are up to six orders of magnitude faster than their exact implementation in large ontologies like SNOMED-CT and GO. Finally, we provide a detailed reproducibility protocol and dataset as supplementary material to allow the exact replication of all our experiments and results.

**Supplementary Information:**

The online version contains supplementary material available at 10.1186/s12859-021-04539-0.

## Background

The development of the gene ontology (GO) [1, 2] has given rise to many significant applications in genomics and proteomics derived from some significant findings that show the correlation of GO-based semantic similarity between genes and proteins with some biological phenomena. For instance, the pioneering work of Lord et al. [3] shows that protein sequence similarity is highly correlated with their corresponding GO-based semantic similarity, which suggests that GO-based similarity measures could be used as protein function prediction tools. Likewise, Freudenberg and Propping [4] show that GO-based similarity measures can be used for the prediction of disease-relevant genes, whilst Sevilla et al. [5] show that gene expression is correlated with GO-based semantic similarity, and Couto et al. [6, 7] show that there is a correlation between the GO-based semantic similarity of proteins and their family similarity based on the Pfam database. As a consequence of these aforementioned findings, a plethora of GO-based semantic similarity measures have been proposed during the last two decades [8–11] which are commonly evaluated in multiple benchmarks [12, 13] using some protein similarity proxies based on their sequence, structure, or common metabolic pathways. Other significant applications of GO-based similarity measures are the prioritization of disease gene candidates [14–16], protein clustering [17], network alignment of protein interaction networks [18], protein functional similarity [19], prediction of the molecular function of genes [20], and characterization of human regulatory pathways [21]. For the reasons above, many software libraries and tools implementing GO-based similarity measures have been proposed in the literature, such as follows: (1) online web tools such as FuSSiMeg [7, 22], G-SESAME [23, 24], FunSimMat [25, 26], Proteinon [27], DaGO-Fun [28], GOssTo [29] and SemSim [30]; (2) R-packages such as GOSim [31] and GOSemSim [32] among others; (3) Python libraries such as FastSemSim [9] and A-DaGO-Fun [33]; and finally, (4) the Java software library called SML [34], which provides an unified and standalone implementation of the most significant ontologies, in addition to set significantly the state-of-the-art for the family of GO-based libraries in terms of performance [34, table 1].

On the other hand, ontology-based semantic similarity measures [35, 36] have been extensively used to estimate the degree of similarity between concepts as perceived by a human being in many text mining and information retrieval (IR) applications, both in the general language domain [35] and the biomedical domain [37, 38]. For instance, ontology-based similarity measures based on Systematized Nomenclature of Medicine Clinical Terms (SNOMED-CT) ontology and the Medical Subject Headings (MeSH) thesaurus have been used in the definition or training of methods for biomedical sentence similarity [39–41], word sense disambiguation [42], estimating the semantic similarity between clinical terms [38] and concepts [43–46], inter-patient distance metrics [47], clinical text classification [48], classification of radiology reports [49], document clustering [50], retrieval of passage for biomedical question answering [51], and article screening [52] among many other applications based on the Unified Medical Language System (UMLS). In order to tackle all aforementioned applications, as well as the growing research interest on the topic, McInnes et al. [53] introduce the first UMLS-based semantic measure library reported in the literature, called UMLS::Similarity (UMLS::Sim), which is implemented as a Perl library together with the standard MySQL database distribution of the UMLS [54] ontologies and vocabularies provided by courtesy of the NLM.^1^

### Main motivation and hypotheses

The main motivation of this work is to overcome some performance and scalability drawbacks in current state-of-the-art semantic measures libraries for the biomedical domain in the fields of biomedical text mining and genomics. Despite the UMLS::Similarity has been extensively used in the literature, it has several significant drawbacks that prevent its use in high-throughput standalone applications, such as a poor performance in the evaluation of measures, as well as a tedious, complex, and long setup process to build several pre-calculated data structures and values stored into an auxiliary database called UMLS::Interface. UMLS::Similarity drawbacks are mainly derived from its use of a scripting programming language like Perl and an ontology representation based on a relational database, which strongly impacts its performance and software architecture. More recently, Harispe et al. [34] introduce the SML Java software library implementing for the first time the most significant ontologies into a single library, such as WordNet [55], SNOMED-CT, MeSH, the Gene Ontology and any others based on the OBO [56] and OWL file formats. However, SML has several significant performance and scalability drawbacks derived from the use of a naive in-memory graph representation based on hash tables and caching, which significantly impacts its overall performance, and very especially, its computation of path-based measures and scalability regarding the ontology size [57, Sect. 1.1.1]. To bridge the aforementioned drawbacks, Lastra-Diaz et al. [57] introduce the HESML Java software library based on WordNet, together with a very efficient and linearly scalable taxonomy representation called PosetHERep that allows the former library outperforms SML by several orders of magnitude [57]. However, the field of biomedical research has not benefited yet from these aforementioned advances because previous HESML versions implement none of the most significant biomedical ontologies, such as SNOMED-CT, MeSH, GO, and others based on the OBO file format. Our main hypothesis is that the efficient and scalable in-memory representation for ontologies provided by HESML should solve these aforementioned performance and scalability drawbacks, as detailed in hypothesis 1 below.

#### **Hypothesis 1**

(H1) A HESML implementation of the main biomedical ontologies should significantly outperform the state-of-the-art biomedical semantic measures libraries in the evaluation of ontology-based semantic similarity measures, such as previously shown for WordNet ontology [57].

The second motivation of our work is to overcome a significant performance and scalability drawback of all path-based semantic similarity measures, which prevents their use in high-throughput experiments, or any practical application demanding their real-time computation. This problem is especially relevant because a recent reproducible survey on word similarity [58–60] shows that one hybrid IC-based similarity measure [35, coswJ&C] sets the state of the art in the family of ontology-based measures for the general domain. However, their practical use in any application is limited because of the lack of an efficient shortest-path algorithm for their real-time computation. Path-based similarity measures require an efficient implementation of any shortest-path algorithm, such as Dijkstra’s algorithm [61]; however, its computational complexity prevents its practical use in high-throughput applications based on large ontologies like SNOMED-CT, GO or WordNet. A common strategy followed by most of the software libraries and tools to tackle the aforementioned problem is to pre-calculate some auxiliary data structures, or all pairwise similarity scores, with the aim of speeding-up the subsequent evaluation of any path-based measure, such as done by UMLS::Similarity, whilst other libraries like SML compute the path-based measures on-the-fly, and store the resulting similarity scores into a cache. The caching of auxiliary data structures and values demands large quantities of memory and complex setup processes, which neither tackle nor solve the main practical problem on the real-time computation of path-based measures at interactive rates, and lead to a poor performance, long setup processes, and running out of memory on large ontologies when they are used on average workstations. Our hypothesis on the aforementioned problem of performance and scalability of path-based similarity measures is that a new approximated shortest-path algorithm, specifically designed for taxonomies, should overcome this problem, as detailed in hypothesis 2 below.

#### **Hypothesis 2**

(H2) A new approximated shortest-path algorithm specifically designed for taxonomies could provide an efficient and linearly scalable method for reformulating and evaluating any path-based semantic similarity measure at interactive rates, whose similarity values would show a high-correlation value as regards its implementation using any exact shortest-path algorithm.

And finally, a third motivation is to provide a larger and most updated set of ontology-based semantic similarity measures and Information Content (IC) models [58, 62] than those provided by UMLS::Similarity and SML libraries, as shown in Tables 2, 3, and 4 .

The aim of this work is to introduce an updated version of the HESML [57] library especially designed for the biomedical domain, called HESML V1R5 [63], together with a fast approximation of the Dijkstra’s algorithm [64] for taxonomies based on a relaxed graph spanner called Ancestors-based Shortest-Path Length (AncSPL), which allows for the first time the real-time computation of any path-based similarity measure on large ontologies, such as SNOMED-CT, GO, and WordNet. HESML V1R5 implements most of the ontology-based similarity measures and IC models reported in the literature as shown in Tables 2, 3 and 4, as well as a very efficient and scalable in-memory representation of WordNet [55], SNOMED-CT, MeSH, GO [1], and other ontologies based on the OBO file format [56]. We introduce a set of reproducible benchmarks for testing our main hypothesis (H1) by comparing the performance of HESML with the UMLS::Similarity and SML libraries on the three most significant biomedical ontologies, as well as several experiments for testing our second hypothesis (H2) as regards the new AncSPL algorithm. Finally, we introduce a reproducibility dataset [65] together with a detailed reproducibility protocol, which is provided as supplementary material (see Additional file 1) to allow the exact replication of all our experiments and results.

## Related work

This section briefly reviews the literature on semantic measures libraries and tools for the biomedical domain, as well as the family of approximated shortest-path algorithms based on graph spanners [66–68], which are related with HESML and our AncSPL algorithm.

### Biomedical semantic measures libraries

The main ontologies used for biomedical text mining and information retrieval applications in health sciences are SNOMED-CT and MeSH, although there are many other ontologies^2^ based on the OBO file format [56]. Nowadays, there are only two semantic measures libraries based on the two aforementioned ontologies as follows: (1) the pioneering Perl software library and online web interface called UMLS::Similarity [53], and (2) the most recent Java software library called SML [34], which introduces several significant contributions, such as a portable and efficient object-oriented language programming, as well as a significant number of methods as shown in Tables 2, 3 and 4, and the implementation for the first time of the most significant biomedical ontologies and WordNet into a single software library, as shown in Table 1. However, both UMLS::Similarity and SML have several significant performance and scalability drawbacks previously detailed in the introduction which encourage our research in this work.

**Table 1:** Ontologies and thesaurus implemented by the three main semantic measures libraries for the biomedical domain

Ontology	UMLS::Similarity	SML	HESML
MeSH	x	x	x
SNOMED	x	x	x
WordNet		x	x
OBO file format		x	x
Gene Ontology		x	x
OWL file format		x	
RDF triples files		x

**Table 2:** Pairwise ontology-based semantic similarity measures implemented by the three main publicly available software libraries for the biomedical domain

	UMLS:: Similarity	SML	HESML
*Gloss-based measures*			
Banerjee and Pedersen [69]	x		
Patwardhan and Pedersen [70], context vector	x		
*Path-based and taxonomy-based measures*			
Rada et al. [71]	x	x	x*
Wu and Palmer [72]		x	x
Wu and Palmer [72] fast (depth-based approximation)	x		x
Leacock and Chodorow [73]	x	x	x*
Stojanovic et al. [74]		x	x*
Maedche and Staab [75]	x		
Zhong et al. [76]	x		
Pekar and Staab [77]	x	x	x*
Li et al. [78], strategy 3			x*
Li et al. [78], strategy 4			x*
Liu et al. [79], strategy 1			x*
Liu et al. [79], strategy 2			x*
Pedersen et al. [44], reciprocal Rada	x		x*
Al-Mubaid and Nguyen [80]	x		x*
Kyogoku et al. [81]		x	
Batet et al. [45]	x		
Hao et al. [82]			x*
Hadj Taieb et al. [83], sim1			x
Hadj Taieb et al. [83], sim2			x
McInnes et al. [84], U-path	x		
*IC-based measures*			
Resnik [85]	x	x	x
Jiang and Conrath [86]	x	x	x
Lin [87]	x	x	x
Schlicker et al. [88]		x	x
Pirró and Seco [89]			x
FaITH [90]	x		x
Garla and Brandt [91]			x
Meng and Gu [92]			x
Gao et al. [93], strategy 3			x
Lastra&García [35], cosJ&C			x
Cai et al. [94], strategy 2			x
*Hybrid IC-based measures*			
Li et al. [ [78] strategy 9			x*
Zhou et al. [95]			x*
Meng et al. [96]			x*
Gao et al. [93], strategy 3			x*
Lastra and García [35], coswJ&C			x*
Lastra and García [35], weigthedJ&C			x*
Cai et al. [94], strategy 1			x*
*Feature-based measures*			
Sánchez et al. [97]	x		x

(*) Real-time reformulation of all path-based measures based on the AncSPL algorithm

On the other hand, most early GO-based software libraries and tools have been implemented as online web tools, such as FuSSiMeg [7, 22], G-SESAME [23, 24], FunSimMat [25, 26], Proteinon [27], DaGO-Fun [28], GOssTo [29] and SemSim [30]. FuSSiMeg [22] introduces the first semantic similarity measure specifically designed for GO terms together with an online web tool for its evaluation, whilst Proteinon [27] provides the first online tool for evaluating GO-based protein semantic similarity. G-SESAME [23, 24] provides a large set of online tools for measuring the semantic similarity between GO terms and the GO-based functional similarity between genes and proteins. FunSimMat [25, 26] provides tools for GO-based protein functional similarity and disease gene prioritization. DaGO-Fun [28] web tool provides a rich set of GO-based similarity measures for GO terms, genes and proteins, as well as tools for the identification of gene and protein candidates for diseases, and tools for gene and protein clustering among others. GOssTo [29] is an online web tool for measuring GO-based similarity between organisms, which implements six similarity measures and it is also distributed as a standalone program based on Java together with an API for developers. SemSim [30] is a web tool which introduces several tools for measuring GO-based similarity between genes and organisms, as well as predicting gene and protein GO annotations, in addition to providing programmatic access to its functionality via Web services. We also find a standalone software called DynGO [98] and other standalone software libraries distributed as R-packages, such as GOSim [31], SemSim [99], GOStats [100], csbl.go [101],  and GOSemSim [32]; Python libraries such as FastSemSim [9] and A-DaGO-Fun [33]; and finally, the aforementioned Java software library called SML [34] which sets the state-of-the-art for the family of GO-based libraries in terms of performance [34, Table 1]. Finally, Le [102] recently introduces a Cytospace [103] app called UFO, which implements a collection of semantic similarity measures and enrichment tools for biomedical ontologies based on the OBO file format.

**Table 3:** Groupwise ontology-based semantic similarity measures implemented by SML and HESML (this work), which are mainly used for genomics applications based on the GO ontology

Groupwise similarity measures	SML	HESML
Maximum [5, formula 2]		x
Average [104, formula 1]		x
Best-Match-Average (BMA) [104, formula 2]		x
SimUI [100]	x	x
SimLP [100]	x	x
SimGIC [105]	x	x
Ali and Deane [18]	x	
Lee et al. [106]	x	
Term Overlap (TO) [107]	x	
Normalized Term Overlap (NTO) [107]	x	
NTO$\_$MAX [107]	x

**Table 4:** Information Content models implemented by the main publicly available software libraries for the biomedical domain

IC models	UMLS ::Similarity	SML	HESML
*Corpus-based IC models*			
Resnik [85, 108]	x	x	x
CPCorpus [62], CPCorpus			x
CPRefCorpus [109],			x
*Intrinsic IC models*			
Seco et al. [110]	x	x	x
Blanchard et al. [111], $IC_{g}$			x
Zhou et al. [112]		x	x
Sebti and Barfroush [113]			x
Sánchez et al. [114]	x	x	x
Sánchez and Batet [115]			x
Meng et al. [116]			x
Harispe et al. [34]		x	x
Yuan et al. [117]			x
Hadj Taieb et al. [118]			x
Adhikari et al. [119]			x
Ben Aouicha and Hadj Taieb [120]			x
Ben Aouicha et al. [121]			x
CondProbHyponyms [62]			x
CondProbUniform [62]			x
CondProbLeaves [62]			x
CondProbCosine [62]			x
CondProbLogistic [62]			x
CondProbRefHyponyms [62]			x
CondProbRefUniform [62]			x
CondProbRefLeaves [62]			x
CondProbRefCosine [62]			x
CondProbRefLogistic [62]			x
CondProbCosineLeaves [62]			x
CondProbRefLogistic-Leaves [62]			x
CondProbRefLeaves-SubsumerRatio [62]			x

#### Shortest-path algorithms based on graph spanners

Our new AncSPL shortest-path algorithm for taxonomies provides an approximated solution for the Single-Source Shortest-Path (SSSP) problem whose aim is to find the shortest-path from a single vertex to the rest of vertexes in a graph. The AncSPL algorithm belongs to the family of approximation methods based on sub-graphs, and it is closely related to the methods based on *graph spanners* whose core idea is to build a simplified version $G'=(V, E')$ of a weighted graph $G=(V,E)$ whose shortest-path distance function satisfies an upper error bound a priori. For this reason, this section focuses on graph spanners. For a comprehensive review of the literature on shortest-path algorithms, we refer the reader to the surveys by Sommer [122], Madkour et al. [123], and Zwick [124].

Graph spanners are pioneering by the works of Peleg and Schaffer [66] and Althofer et al. [67], whilst the current state-of-the-art spanner construction algorithm is introduced by Elkin and Solomon [68]. Given a graph $G=(V,E)$, a sub-graph $G'=(V, E')$ is a t-spanner if for every vertex pair $u,v \in V$ the distance in the sub-graph $d_{G'}(u,v)$ is at most t times longer than the distance $d_{G}(u,v)$ in *G*, such that $\forall u,v \in V, d_{G'}(u,v) \le t \cdot d_G(u,v)$. Spanner-based algorithms are based on well-founded theoretical results in graph theory, in addition to be of great practical value in many scenarios. However, they have two drawbacks in the context of our problem as follows. On the one hand, graph spanners have a high complexity derived from the need for computing a spanning graph considering all graph vertexes, and on the other hand, they do not take advantage of the knowledge of the graph structure in special cases such as the single-root taxonomies considered herein. Elkin and Solomon [68] point that “the only algorithms for constructing sparse and lightweight spanners for general graphs admit high running times”. Precisely, we propose AncSPL to take advantage of the intrinsic structure of the single-root taxonomies to provide an efficient approximation SSSP algorithm.

## Implementation

This section is divided into two parts as follows. First part introduces the new semantic measures library for the biomedical domain, called HESML V1R5, whilst the second part introduces a real-time algorithm for the computation of the shortest-path between concepts in large ontologies, called AncSPL, whose performance and approximation quality are tested in our experiments.

### The new semantic measures library

HESML V1R5 is a new version of the HESML [57] open-source Java software library that extends its applicability to the biomedical domain by implementing the SNOMED-CT, MeSH, GO [1, 2], and OBO file format ontologies [56], in addition to WordNet [55]. HESML V1R5 is a self-contained Java software library of pairwise and groupwise ontology-based semantic similarity measures, and information content (IC) models, which also supports the evaluation of pre-trained word embedding models in three different file formats. The core innovation of HESML is a very efficient and linearly scalable in-memory representation for taxonomies, called PosetHERep, which was introduced in the first version of HESML [57] based on WordNet. PosetHERep is mainly responsible for the real-time performance and scalability with low memory consumption shown by HESML. PosetHERep converts HESML V1R5 into the most efficient, scalable, and portable semantic measures library reported in the literature, as shown by the benchmarks based on WordNet and large synthetic ontologies reported in [57], and the benchmarks on biomedical ontologies evaluated in this work. For more information on the data structures and algorithms of the PosetHERep representation model, we refer the reader to [57, Sect. 3.2].

HESML V1R5 implements the largest set of pairwise ontology-based semantic measures and IC models reported in the literature, as shown in Tables 2 and 4 respectively. However, this first version of HESML for the biomedical domain does not include some specific GO-based pairwise and groupwise similarity measures which will be included in forthcoming versions. Likewise, HESML V1R5 provides for the first time real-time reformulations for most of the path-based and hybrid IC-based measures reported in the literature, which are based on the new AncSPL shortest-path algorithm introduced herein.

**Fig. 1:**
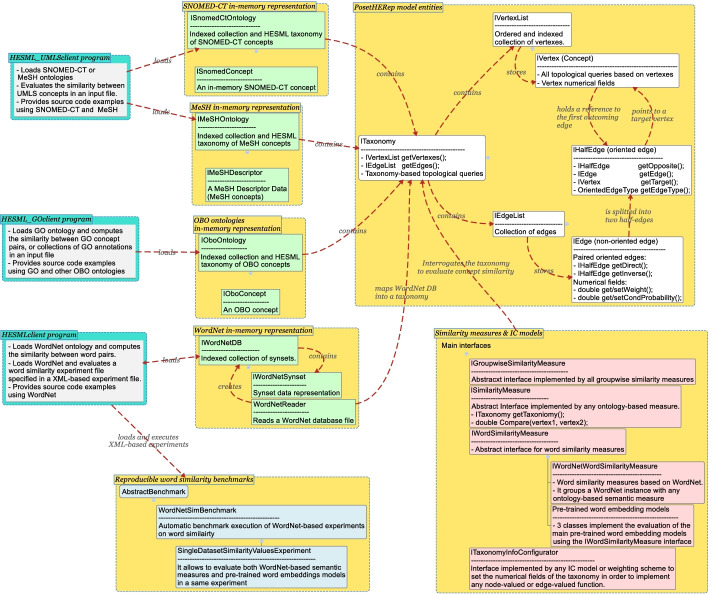
HESML V1R5 architecture showing the main functional blocks and abstract interfaces. Boxes in yellow show main abstract objects and interfaces contained in the HESML library, whilst boxes in turquoise blue show main HESML client programs, whose aim is to evaluate semantic similarity measures implemented in HESML on the SNOMED-CT, MeSH, GO, and WordNet ontologies

HESML V1R5 is a self-contained evaluation and experimentation platform on word and concept similarity and relatedness, which is especially well suited to run large experimental surveys by supporting the execution of automatic reproducible experiment files based on different XML-based file formats. Despite HESML V1R5 implements the most significant ontologies reported in the literature, it could also be easily extended to manage other ontology file formats, such as OWL or RDF files, by implementing the proper parsers as detailed in [57]. HESML V1R5 library has been completely developed in NetBeans 8 and Java 8, being distributed with three WordNet versions and GO. HESML V1R5 integrates some complementary Java console programs shown in turquoise blue boxes in Fig. 1, which use the HESML core library to run reproducible experiments and evaluate the semantic similarity between words, UMLS concepts, or GO terms and GO annotation sets (genes and proteins) which are based on WordNet, SNOMED-CT or MeSH, and GO.

*HESML Software Architecture.* Figure 1 shows a concept map detailing the HESML V1R5 architecture. The core HESML component is the half-edge taxonomy representation (PosetHERep) defined by the yellow entities within the largest box in yellow. Red entities in the block entitled ‘Similarity measures & IC models’ represent the interfaces that should be implemented to define new methods, such as general groupwise (IGroupwiseSimilarityMeasure) or pairwise (ISimilarityMeasure) similarity measures, word similarity measures (IWordSimilarityMeasure) including pre-trained word embedding models, or new IC models (ITaxonomyInfoConfigurator). Every type of ontology is implemented by a specific collection of Java classes and interfaces which holds a ITaxonomy object to represent its corresponding ontology, such as the ISnomedCtOntology, IMeSHOntology, IOboOntology and IWordNetDB interfaces shown in Fig. 1. All the HESML objects are provided as Java interfaces, being instanced by factory objects not represented in the figure above. For a detailed introduction to the software architecture, PosetHERep, and main algorithms of HESML, we refer the reader to its introductory paper [57], and the HESML web page.^3^

*Current methods implemented by HESML.* Table 1 shows the ontologies and ontology-based file formats implemented by the three main semantic measures libraries for the biomedical domain evaluated herein, whilst Tables 2, 3, and 4 shows the pairwise and groupwise ontology-based semantic similarity measures, and the IC models, implemented by the aforementioned software libraries respectively. Finally, Table 5 shows a collection of pre-trained word embedding models which were evaluated in a large benchmark [58] on word similarity using three new HESML classes called EMBWordEmbeddingModel, UKBppvWordEmbeddingModel and NasariWordEmbeddingModel respectively, which implement the evaluation of the (*.emb), (*.ppv) UKB [125] and Nasari [126] word vector file formats. Thus, HESML is able to evaluate both semantic similarity measures based on any ontology shown in Table 1 and recent word embedding models in a common software platform.

**Table 5:** Collection of pre-trained word embedding (WE and WEC) models and ontology-based vector models (OVM) evaluated in a previous series of experiments [58–60] by using the Java classes implementing their evaluation

WN	Family	Word embedding model
Yes	WEC	Attract-repel [127]
No	WE	FastText [128]
No	WE	GloVe [129]
No	WE	CBOW [130]
Yes	WEC	SymPatterns (SP-500d) [131]
No	WEC	Paragram-ws [132]
No	WEC	Paragram-sl [132]
Yes	WEC	Counter-fitting (CF) [133]
Yes	OVM	WN-RandomWalks [134]
Yes	OVM	WN-UKB [125]
Yes	OVM	Nasari [126]

First column details which methods use WordNet during their training

*Extending the HESML functionality.* HESML can be extended in different directions by developing new features as follows: (1) further pairwirse or groupwise semantic similarity measures; (2) further IC models; (3) further ontology parsers for unimplemented ontology file formats; (4) further evaluators for unimplemented pre-trained word embedding models or file formats; (5) further client programs dealing with specific ontologies; and (6) further new tools based on ontology-based semantic similarity measures, such as gene clustering and other gene enrichment tools, or sentence similarity measures among many other text mining applications. For instance, in order to develop any new similarity measure, you should develop a class, which implements the appropriate interface, by following any of the multiple source code examples in the library, then the reader should include its creation in its corresponding factory function in the class *MeasureFactory*. In order to develop any new IC model, the reader should develop a class implementing the *ITaxonomyInfoConfigurator* by deriving from *AbstractICmodel* class. Finally, HESML source code is clear and well documented, thus the readers will find a lot of source code examples to learn the HESML basics on its use and extension. In addition, the readers can subscribe to the HESML community forum, or contact the authors, as detailed in the availability section.

### The new shortest-path algorithm for taxonomies

Our new shortest-path algorithm for taxonomies, called ancestors-based shortest-path length (AncSPL), is a fast approximation of the Dijkstra’s algorithm that is based on a min-priority queue implementation [61] constrained to a sub-graph derived from the ancestor sets of the source and target concepts. AncSPL uses an exact shortest-path algorithm that runs on the sub-graph derived from the ancestor sets by ignoring those edges connecting to any node not belonging to the sub-graph; thus, AncSPL does not require any graph transformation or auxiliary data structure. Implementation of the Dijkstra’s algorithm in HESML is very efficient because PosetHERep [57] allows traversing any taxonomy in linear time as regards the number of edges. In addition, the AncSPL algorithm is easy to implement, all topological queries required are efficiently computed by HESML and it does not require any complex auxiliary data structure or preprocessing as required by the most of approximated SSSP methods for general graphs.

Given a single-root taxonomy ${\mathcal {C}} = (C, \le _C, \Gamma )$, where $(C, \le _C)$ is a partially ordered set, and $\Gamma \in C$ is a distinguished supreme element called the root, such that $\forall c_i \in C \rightarrow c_i \le _C \Gamma$. The core idea and underlying hypothesis of our AncSPL algorithm is that given two randomly selected taxonomy nodes $c_i,c_j \in C$, most of the shortest paths between them will be contained in a set defined by the union of their ancestor sets. Our aforementioned underlying hypothesis is always true on any tree-like taxonomy, such as MeSH, in whose case we can use a direct, exact, and linearly scalable formula (line 5, Algorithm 1) to compute the length of the shortest path. However, this later formula is not exact for general taxonomies with multiple inheritance, such as WordNet, SNOMED-CT, and GO.

Our new AncSPL algorithm is detailed in Algorithm 1 box. PosetHERep representation [57] implemented by HESML allows that all topological queries involved in the implementation of AncSPL can be efficiently computed in linear time as regards each node depth value, such as the computation of the lowest common subsumer (LCS) concept, concept depth, and ancestor sets. For this reason, the combination of fast topological queries provided by HESML together with a large graph reduction based on the ancestor sets allows getting a very efficient approximation of the exact value for the length of the shortest path between concepts in any non-tree-like taxonomy. Finally, we refer the reader to the *Vertex.getFastShortestPathDistanceTo()* method in HESML V1R5 [63] to see our current implementation of AncSPL. Likewise, we provide the definition of the LCS function used in step 5 of AncSPL, and the HESML min-priority queue implementation of the Dijkstra’s algorithm in Algorithm 2 and 3 boxes, respectively.

**Fig. 2:**
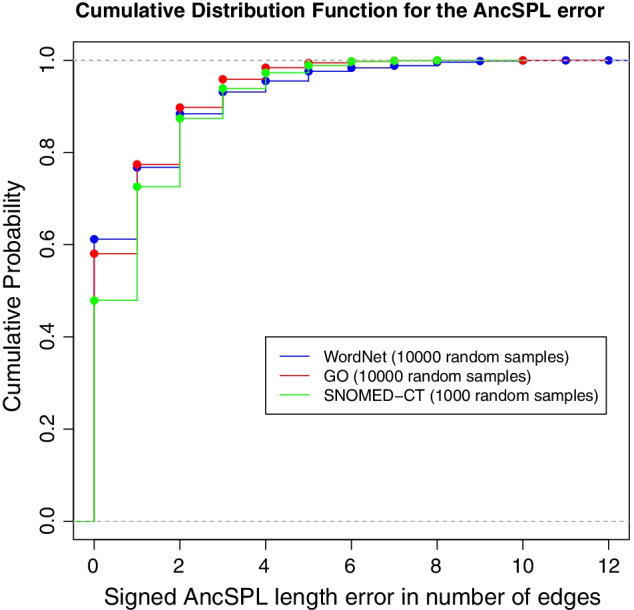
This figure shows the cumulative distribution function (CDF) of the signed AncSPL length error function $E(c_i,c_j) = AncSPL(c_i,c_j) - spl(c_i,c_j)$, where $spl(c_i,c_j)$ is the exact length of the shortest path between concepts $c_i$ and $c_j$ in SNOMED-CT, GO, and WordNet ontologies

*Approximation error of AncSPL.* The shortest-path length estimated by AncSPL is always greater or equal than the exact value, it means that let be $spl(c_1,c_2)$ the exact length value between concepts $c_1$ and $c_2$, then $AncSPL(c_1,c_2) \ge spl(c_1,c_2)$ for any concept pairs in any ’is-a’ taxonomy, as shown in Fig. 2 for SNOMED-CT, GO, and WordNet ontologies, respectively. Consequently, the AncSPL reformulation of any path-based similarity measure will always return a less or equal similarity value than their corresponding exact version. On the other hand, $AncSPL(c_1,c_2)$ will be equal to $spl(c_1,c_2)$ when either the shortest path between both concepts is contained in the common ancestor set or the taxonomy is a tree. Thus, any AncSPL reformulation will return the same value that the original path-based measure in these latter cases, and for tree-like taxonomies as MeSH, any AncSPL reformulation will be exact for any concept pair by definition. 
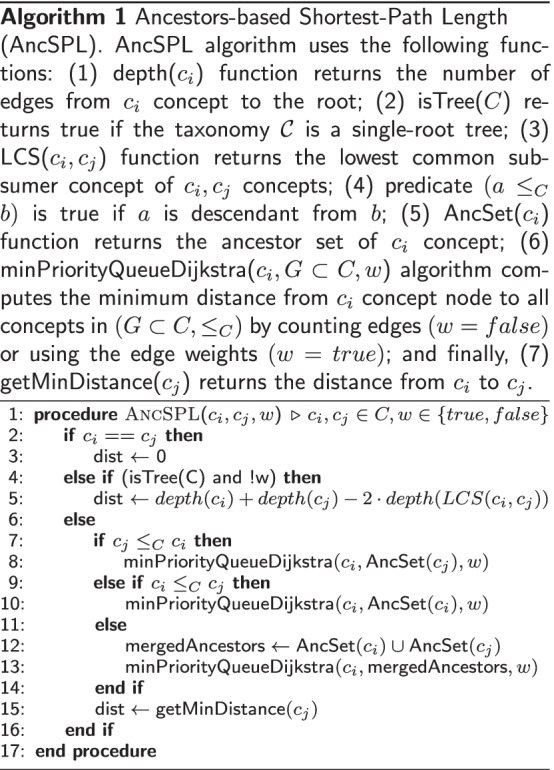


#### Time complexity of the AncSPL algorithm

AncSPL uses two different methods to compute the length of the shortest path between concepts as follows: (1) an exact method for tree-like taxonomies defined in step 5 of Algorithm 1, which is based on the LCS function detailed in Algorithm 2; and (2) a min-priority queue implementation of the Dijkstra’s algorithm constrained to the ancestors-based subgraph defined in steps 7–14 of Algorithm 1, which is based on the efficient PosetHERep representation introduced by HESML [57] and a Java PriorityQueue object, as detailed in Algorithm 3. 
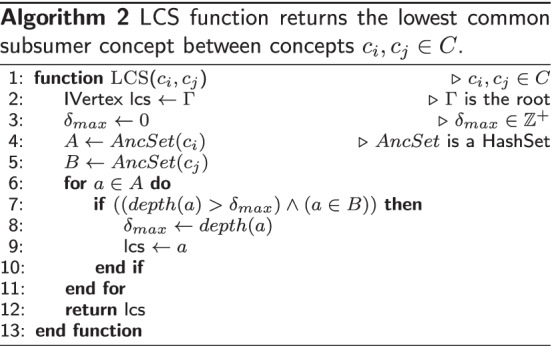


The Java PriorityQueue class uses a priority heap whose time complexity is *O*(*log*(*n*)) for the insertion (add) and poll operations, and *O*(*n*) for the remove operation, as pointed out in its user’s documentation.^4^ Thus, the time complexity of the AncSPL algorithm detailed in Algorithm 1 box can be elucidated by directly inspecting the auxiliary function and procedure detailed in Algorithm 2 and 3 boxes, respectively. 
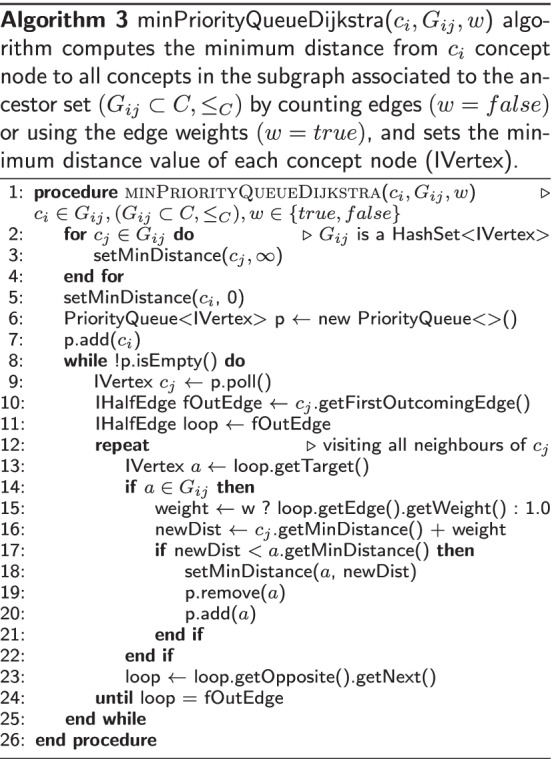


##### Theorem 1

*Let be a single-root taxonomy* ${\mathcal {C}} = (C, \le _C, \Gamma )$, *where* $(C, \le _C)$ *is a partially ordered set, and* $\Gamma \in C$ *is a distinguished supreme element called the root, such that* $\forall c_i \in C \rightarrow c_i \le _C \Gamma$, *and let be* $(G_{ij} \subset C, \le _C, \Gamma )$ *a sub-taxonomy of* ${\mathcal {C}}$ *made up by the common ancestor set of concepts* $c_i,cj \in C$, *such that* $G_{ij} = AncSet(c_i) \bigcup AncSet(c_j)$, *where* $AncSet(x)=\{c \in C, x \le _C c\}$. *Then, the time complexity of the AncSPL algorithm is linear in the dimension of the sub-taxonomy with* *O*(*N*), being $N =|G_{ij}|$ *the dimension of the common ancestor-based sub-taxonomy* $G_{ij}$.

##### *Proof*

There are two cases and workflows for the execution of AncSPL depending on the input taxonomy is tree-like (case 1) or not (case 2). Thus, time complexity of AncSPL denoted by $TC_{AncSPL}$ will be equal to the time complexity of the Algorithm 2 ($TC_2$) or the Algorithm 3 ($TC_3$) as proven below.

(Case 1) For tree-like taxonomies processed in step 5, AncSPL evaluates the sorthest-path length by computing the distance to the Lowest Common Subsummer (LCS) using the Algorithm 2 whose time complexity can be computed as follows:

1. Steps 2–3 takes 2 operations in constant time $k_1$.
2. Ancestor sets in steps 4–5 can be obtained either in 2 operations in constant time $k_2$ if they are cached, or $O(k_3N)$ otherwise by retrieving the ancestor nodes using PosetHERep [57], where $N=|AncSet(c_i)|\le |G_{ij}|$ is the number of ancestors of $c_i$.
3. Loop in steps 6–11 is executed *N* times.
4. Step 7 takes 3 operations in constant time $k_4$.
5. Steps 8–9 take 2 operations in constant time $k_5$.

Thus, summing the overall time consumed by all steps detailed above, and considering that the ancestor sets can be cached, time complexity of Algorithm 2 is as follows:

$$\begin{aligned} TC_2 = {\left\{ \begin{array}{ll} O(k_1 + k_2 + (k_4 + k_5)N) = O(kN)\text {, if cached} \\ O(k_1 + (2k_3 + k_4 + k_5)N) = O(kN)\text {, otherwise} \end{array}\right. }\end{aligned}$$

(Case 2) For non-tree-like taxonomies processed by the else-branch in step 6, AncSPL computes the shortest-path length using the Algorithm 3 with the sub-taxonomy $G_{ij}$ as input. Thus, let be $N = |G_{ij}|$ the number of common ancestor nodes, then its time complexity can be computed as follows:

1. Steps 2–5 takes exactly $N + 1$ operations in constant time $k_1$, it means $O(k_1(N + 1))$ time.
2. Steps 6–7 takes 2 operations in constant time $k_2$
3. Traversing loop in steps 8–25 is executed *N* times.
4. Step 9 requires *O*(*log*(*n*)) time, being *n* the current item count stored within the priority queue. However, in step 9, the queue mainly stores the adjacent nodes of the last visited node in each iteration. Thus, the time will be $O(k_3log({\bar{E}}_{G_{ij}})$ in average, where ${\bar{E}}_{G_{ij}}$ is the average number of adjacent nodes per ancestor for each node $c_i \in G_{ij}$.
5. Loop in steps 12–24 is executed $E^j_{G_{ij}}$ times $\forall c_j \in G_{ij}$, where $E^j_{G_{ij}}$ is the number of adjacent nodes of $c_j$ contained in the sub-taxonomy $G_{ij}$.
6. Step 14 takes 1 operation in constant time $k_4$.
7. Steps 15–18 takes constant time $k_5$.
8. Step 19 takes *O*(*n*) time for removing the visited node *a*, being *n* the current item count stored within the queue. However, using the same argument provided in step 3.1 above, the time will be $O(k_6{\bar{E}}_{G_{ij}})$ in average.
9. Step 20 requires *O*(*log*(*n*)) time for inserting the visited node *a*, but using the same argument above, the time will be $O(k_7log({\bar{E}}_{G_{ij}}))$ in average.
10. Step 23 takes 2 operations in constant time $k_8$

Thus, summing the overall time consumed by all steps of Algorithm 3 detailed above, its time complexity $(TC_3)$ is:

$$\begin{aligned} TC_3&= O(k_1(N + 1) + k_2 + N(k_3log({\bar{E}}_{G_{ij}}) \\&\quad + {\bar{E}}_{G_{ij}}(k_4 + k_5 + k_6{\bar{E}}_{G_{ij}} + k_7log({\bar{E}}_{G_{ij}}) + k_8))) \\&= O(k_1(N + 1) + k_2 + N(k_3log({\bar{E}}_{G_{ij}}) \\&\quad + k_9{\bar{E}}_{G_{ij}} + k_6{\bar{E}}^2_{G_{ij}} + k_7{\bar{E}}_{G_{ij}}log({\bar{E}}_{G_{ij}}))) \\ \end{aligned}$$

because $\forall x \ge 2 \Rightarrow x^2>> xlog(x) > log(x)$ we can approximate $TC_3$ as follows:

$$\begin{aligned} TC_3&= O((k_1 + k{\bar{E}}^2_{G_{ij}})N + k_1 + k_2) \\&= O((k_1 + k{\bar{E}}^2_{G_{ij}})N + k') \\&= O(k{\bar{E}}^2_{G_{ij}}N) \\ \end{aligned}$$

$$\square$$

##### Corollary 1

*Let be a single-root taxonomy* ${\mathcal {C}} = (C, \le _C, \Gamma )$ *as defined in theorem above,* $c_i,c_j \in C$ *two arbitrary distinct concepts,* ${\bar{E}}_C$ *is the average number of adjacent nodes* $\forall c \in C$, and $N_{max}$ *is the maximum number of ancestor nodes for any concept* $c_i \in C$. *Then, the time complexity* ($TC_{AncSPL}$) *is upper bounded as follows:*

$$\begin{aligned} TC_{AncSPL} \le {\left\{ \begin{array}{ll} kN_{max}, \quad \quad \quad \text {C is tree-like} \\ k{\bar{E}}^2_CN_{max}, \quad \text {otherwise} \end{array}\right. } \end{aligned}$$

##### *Proof*

The proof of the corollary follows directly from the proof of the theorem above. $\square$

**Fig. 3:**
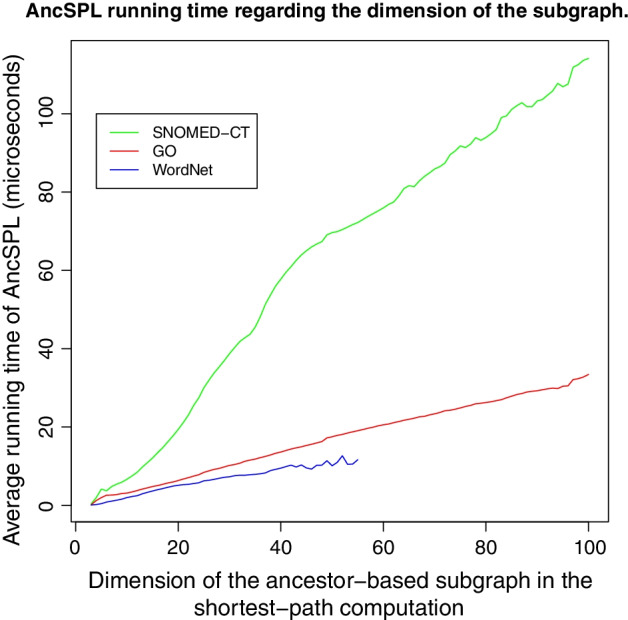
This figure shows the average running time in micro seconds ($\mu$s) obtained in evaluating the AncSPL-Rada similarity measure for groups of at least $10^6$ random concept pairs in SNOMED-CT and GO, and at least $10^7$ random pairs in WordNet, which are grouped by the dimension of their corresponding ancestor-based subgraph

The dimensions of the largest ancestor sets $(N_{max})$ for the ontologies evaluated herein are as follows: $N^{SND}_{max}=129$ , $N^{GO}_{max}=98$, $N^{MSH}_{max}=14$, and $N^{WN}_{max}=35$. The performance of AncSPL is much higher on MeSH than the remaining ontologies because, on the one hand, its $N_{max}$ value is significantly lower than the corresponding value of the remaining ontologies, and on the other hand, the AncSPL time complexity is much lower for tree-like ontologies than for non-tree-like ones because $TC_2$ linearly depends on *kN*, whilst $TC_3$ depends on $k{\bar{E}}^2_{G_{ij}}N$. Thus, the intrinsic feature ${\bar{E}}^2_{G_{ij}}$ scales the time complexity of AncSPL on non-tree-like ontologies, as shown in Fig. 3.

#### Reformulating any path-based similarity measure

Any path-based semantic similarity or distance measure can be reformulated using the AncSPL algorithm by substituting the call to the function *spl* computing the exact length of the shortest path between concepts by a call to the *AncSPL* function. For example, formulas (1–2) show the AncSPL reformulation of the reciprocal Rada et al. distance [71], called $sim_{path}$ [44], whilst formulas (3–4) show the reformulation of the Leacock-Chodorow [73] similarity measure.

1 $$\begin{aligned} sim_{path}(c_1,c_2)&= \frac{1}{1 + spl(c_1,c_2)} \end{aligned}$$

2 $$\begin{aligned} sim_{AncSPL-path}(c_1,c_2)&= \frac{1}{1 + {\scriptstyle AncSPL}(c_1,c_2)} \end{aligned}$$

3 $$\begin{aligned} sim_{L \& C}(c_1,c_2)&= -log\left( \frac{1 + spl(c_1,c_2)}{2 \times {\scriptstyle maxDepth}}\right) \end{aligned}$$

4 $$\begin{aligned} sim_{\scriptscriptstyle AncSPL-L \& C}(c_1,c_2)&= {\scriptstyle -log}\left( \frac{1 + {\scriptstyle AncSPL}(c_1,c_2)}{2 \times maxDepth}\right) \end{aligned}$$

## Results

This section introduces a series of reproducible experiments whose main goals are as follows: (1) to test our main hypothesis H1 by evaluating and comparing the performance of the new HESML V1R5 library with the state-of-the-art biomedical semantic measure libraries based on the main biomedical ontologies; and (2) to test our second hypothesis H2 on the new AncSPL shortest-path algorithm introduced in this work. All experiments reported herein were implemented in an Ubuntu 20.04 desktop based on one AMD Ryzen 7 5800x CPU (16 cores) with 64 Gb RAM and 2TB Gb SSD disk. Likewise, we provide a very detailed reproducibility protocol and dataset as supplementary material to allow the exact replication of all experiments and results introduced herein (see Aditional file 1).

*Evaluation of HESML performance.* We compare the performance of HESML V1R5 with UMLS::Similarity 1.47 and SML 0.9 libraries, which are the only publicly available semantic measures libraries for SNOMED-CT and MeSH, whilst SML is also the best performing semantic measures library based on GO (see [34, Table 1]). First, we evaluate the average speed of each library, measured in concepts by second, in the evaluation of the semantic similarity of a sequence of randomly generated pairs of UMLS or GO concepts using the SNOMED-CT, MeSH, and GO ontologies as shown in Tables 6, 7 and 8 respectively. Next, we evaluate the average speed of each library, measured in sentences by second as shown in Table 9, in the evaluation of the similarity of a subset of 30 sentence pairs extracted from the MedSTS [135] sentence similarity benchmark, and 1 million sentence pairs extracted from the BioC corpus [136], by implementing the UBSM [39] sentence similarity measure in combination with some ontology-based semantic similarity measures based on MeSH. Table 9 also reports the average speed measured in UMLS Concept Unique Identifier (CUI) pairs per second to compare the results reported for the evaluation of either 30 sentence pairs or 1 million.

*Selection of ontology-based similarity measures.* We use the Rada et al. [71], Lin [87] and Wu and Palmer [72] similarity measures as a common representative sample to evaluate the performance of the three aforementioned libraries in all our experiments. However, we exclude the evaluation of the Wu-Palmer measure for the SML library because it does not provide the same depth-based version implementation than HESML or UMLS::Similarity. We selected these three similarity measures mentioned above because of several reasons. Firstly, they are implemented by the three libraries analyzed herein, as shown in Table 2. Secondly, Rada et al. measure is a good representative for the family of path-based similarity measures, whilst Lin and Wu-Palmer measures are good representatives for the families of similarity measures based on IC models and taxonomic features, respectively. Third, these three later measures allow evaluating the HESML performance in three graph-based algorithms used by most of ontology-based similarity measures as follows: (1) the computation of the length of the shortest path between concepts; (2) the computation of the Most Informative Common Ancestor (MICA) concept; and (3) the Lowest Common Subsumer (LCS) concept. Fourth, IC-based measures based on a single computation of the MICA concept will exhibit the same performance, such as the measures by Resnik [85], Lin [87], and Jiang-Conrath [86], whilst all path-based using a single computation of the length of the shortest path between concepts will also share the same performance. Finally, current authors showed theoretically [109, Table 3] and experimentally that many ontology-based similarity measures reported in the literature are based on monotone transformations or reformulations of other path-based or IC-based measures. For all the reasons above, the performance results reported herein could be extrapolated to other similar measures based on the same set of graph-based algorithms.

*Experimental setup.* All our experiments were generated by running a Java console program called HESML_UMLS_benchmark on a Docker container based on UBUNTU 20.04, as detailed in Appendix A (see Additional file 1), which is provided as supplementary material [65] to allow the exact replication of all experiments and results introduced herein. Because there are large differences in the average speed of each library, especially UMLS::Similarity, we used a different number of concept pairs (samples) per library from the same randomly-generated sequence of UMLS concept (CUI) pairs. Our reproducibility dataset [65] also provides the raw data files obtained in three runs of our experiments. All experiments reported herein are based on HESML V1R5.0.2 release, which is publicly available at HESML GitHub repository^5^ and its permanent dataset [63].

*Testing our hypothesis for the AncSPL algorithm.* Concerning the new AncSPL algorithm, we include the evaluation of the AncSPL-Rada reformulation of the Rada et al. [71] measure in Tables 6, 7, 8 and 9 to compare the performance of the AncSPL-based measures with that obtained by their exact implementations. Finally, to test the second part of our hypothesis H2 on the approximation quality of our AncSPL algorithm, we evaluate the Pearson and Spearman correlation values between the similarity values returned by a set of path-based similarity measures for 50, 100, 200, and 1000 random CUI pairs in SNOMED-CT, GO, and WordNet non-tree-like ontologies and those values returned by their reformulation based on the AncSPL algorithm, as shown in Table 10.

*Approximation error of AncSPL.* To analyze the absolute approximation error made by AncSPL in the estimation of the exact shortest-path length on non-tree-like ontologies, Fig. 2 shows the cumulative distribution function (CDF) for a set of random samples of the signed shortest-path length error measured in number of edges in SNOMED-CT, GO, and WordNet.

*Testing the AncSPL time complexity.* To test experimentally the time complexity of AncSPL, Fig. 3 reports the average running time obtained in evaluating the AncSPL-Rada similarity measure on groups of random concept pairs grouped by the dimension of their corresponding ancestor-based subgraph in SNOMED-CT, GO, and WordNet ontologies, respectively. These experiments evaluate the time complexity of the AncSPL algorithm on non-tree-like taxonomies based on the min-priority queue implementation of the Djikstra’s algorithm 3 using the PosetHERep taxonomy representation [57], when the input graph is constrained to the corresponding ancestor-based subgraph defined by the AncSPL algorithm 1. Every running time value is measured by evaluating at least $10^6$ random concept pairs per group in SNOMED-CT and GO, and at least $10^7$ pairs per group in WordNet. Likewise, to test experimentally the impact of the intrinsic scaling factor $k{\bar{E}}^2_{G_{ij}}$, which scales the linear time complexity of AncSPL in non-tree-like ontologies as defined by $TC_3$, Table 12 compares the theoretical and experimental values for the expected running-time ratios between ontologies derived from the average number of adjacent nodes per ancestor set ${\bar{E}}_{C}$ measured on the ontologies.

*Large GO-based similarity evaluation.* To show the performance of HESML in a large high-demanding GO-based similarity task, Table 11 shows the performance of four groupwise GO-based similarity measures in the evaluation of the pairwise protein similarity between all proteins of the Homo Sapiens and Canis lupus familaris organisms, using their corresponding protein^6^ files in GO annotation file (GAF) file format.

*Evaluating HESML real-time capabilities.* The performance of real-time applications is measured as the time in which an application should answer to a pre-defined event. The main functionality provided by HESML is the capability to evaluate on-the-fly the semantic similarity between ontology concepts at very high rates measured in concept pairs per second without costly auxiliary data structures, as shown in Tables 6, 7 and 8. This later functionality can be used in other ontology-based semantic similarity tasks, such as the evaluation of biomedical sentence similarity reported in Table 9, or the evaluation of GO-based protein similarity reported in Table 11, among others. Thus, HESML allows the proposal of new real-time biomedical applications demanding either a large number of ontology-based semantic similarity evaluations in a pre-defined fraction of a second or the capability to process large ontology-based annotated data files in a pre-defined time as a measure of their quality of service.

**Table 6:** Average speed in CUI concept pairs per second (pairs/s) for the evaluation of random CUI pairs with three representative ontology-based similarity measures based on the SNOMED-CT US 2019AB ontology (357,406 nodes) implemented by the three UMLS-based semantic measures libraries reported in the literature

Similarity measure	UMLS::Similarity	SML	HESML
	Avg. speed (pairs/s)	Avg. speed (pairs/s)	Avg. speed (pairs/s)
Rada [71]	**0.122** (15)	xxx	0.041 (15)
AncSPL-Rada (this work)	–	–	**30110** $(10^7)$
Lin-Seco [87, 110]	0.744 (500)	202160 $(10^7)$	**491942** $(10^7)$
Wu-Palmer$_{fast}$ [72]	0.035 (15)	–	**435252** $(10^7)$

Best performing values are shown in bold. Non-implemented methods (–) or more than 1 h/pair (xxx). UMLS::Similarity uses caching for the shortest path computations. The number of random CUI pairs evaluated to measure each value is shown between parentheses

**Table 7:** Average speed in CUI concept pairs per second (pairs/s) for the evaluation of random CUI pairs with three representative ontology-based similarity measures based on the MeSH ontology (Nov, 2019. 59,747 nodes) implemented by the three UMLS-based semantic measures libraries reported in the literature

Similarity measure	UMLS::Similarity	SML	HESML
	Avg. speed (pairs/s)	Avg. speed (pairs/s)	Avg. speed (pairs/s)
Rada [71]	30.43 (15)	0.096 (15)	**644729**$(10^7)$
AncSPL-Rada (this work)	–	–	**705189**$(10^7)$
Lin-Seco [87, 110]	140.82 (500)	532913$(10^7)$	**824307**$(10^7)$
Wu-Palmer$_{fast}$ [72]	21.34 (15)	–	**717535**$(10^7)$

Best performing values are shown in bold. Non-implemented methods (–). The number of random CUI pairs evaluated to measure each value is shown between parentheses

**Table 8:** Average speed in GO concept pairs per second (pairs/s) for the evaluation of two representative ontology-based similarity measures based on the Gene Ontology [1, 2] (2020-05-02 version, 44509 nodes)) implemented by state-of-the-art SML [34] library and HESML

Similarity measure	Measure type	SML	HESML
		Avg. speed (pairs/s)	Avg. speed (pairs/s)
Rada [71]	Edge-counting	0.077 (20)	**3.217** (20)
AncSPL-Rada (this work)	Edge-counting	–	**140422** $(10^7)$
Lin-Seco [87, 110] IC model	IC-based	372140 $(10^7)$	**1063219** $(10^7)$

Best performing values are shown in bold. The number of random GO concept pairs evaluated to measure each value is shown between parentheses

**Table 9:** Average speed in sentence pairs per second (sent/s) and CUI pairs per second (CUIs/s) for the evaluation of the UBSM [39] sentence similarity measure combined with three representative ontology-based similarity measures based on MeSH (Nov, 2019) in 30 sentence pairs extracted from the MedSTS [135] sentence similarity dataset, and 1 million sentence pairs extracted from BioC corpus [136]

Pairwise sentence comparison based on MeSH	UMLS::Sim (30 pairs)		SML (30 pairs)		HESML (30 pairs)		${{HESML\, (10^6\, pairs)}}$	
Similarity measure	Avg. speed (sent/s)	Avg. speed (CUIs/s)	Avg. speed (sent/s)	Avg. speed (CUIs/s)	Avg. speed (sent/s)	Avg. speed (CUIs/s)	Avg. speed (sent/s)	Avg. speed (CUIs/s)
Rada et al. [71]	0.441	36.63	0.126	10.478	**2830.189**	235000	7982.222	337843.826
AncSPL-Rada (this work)	–	–	–	–	**2542.373**	211101.695	7958.742	336850.041
Lin-Seco [87, 110]	0.782	64.956	2586.207	214741.379	**3125**	259479.167	8166.185	345629.98
Wu-Palmer$_{fast}$ [72]	0.181	15.067	–	–	**3125**	259479.167	7892.959	334065.805

We provide the average evaluation in normalized CUI pairs per second to allow a fair and unbiased comparison of the results reported for 30 and 1 million sentence pairs. The dataset with 30 sentence pairs requires 2491 pairwise CUI comparisons, whilst the 1 million sentence pairs dataset requires 42324534 pairwise CUI comparisons. Best performing values are shown in bold. Non-implemented methods (–)

**Table 10:** This table shows the Pearson (r) and Spearman ($\rho$) correlation values between the similarity values returned by a set of path-based similarity measures and those values returned by their reformulation based on the new AncSPL algorithm for a sequence of 1000 random CUI pairs in SNOMED-CT 2019AB, GO (2020-05-02), and WordNet 3.0

Base measure	AncSPL reformulation	50 samples		100 samples		200 samples		1000 samples	
		r	$\rho$	r	$\rho$	r	$\rho$	r	$\rho$
Correlation values in SNOMED-CT ($\text {tree-like}_{\sigma }$ = 0.425)									
Rada [71]	AnsSPL-Rada	0.9214	0.9412	0.9413	0.9444	0.9357	0.9352	0.9231	0.9217
Leacock and Chodorow [73]	AnsSPL-Leacock	0.9409	0.9412	0.9479	0.9444	0.9422	0.9352	0.9217	0.9217
coswJ&C [35]	AnsSPL-coswJ&C	0.9136	0.9506	0.9583	0.9747	0.9761	0.9775	0.941	0.9714
Correlation values in GO ($\text {tree-like}_{\sigma }$ = 0.446)									
Rada [71]	AnsSPL-Rada	0.8571	0.8277	0.9133	0.9085	0.8883	0.8868	0.9074	0.8947
Leacock and Chodorow [73]	AnsSPL-Leacock	0.8542	0.8277	0.9109	0.9085	0.9007	0.8868	0.9191	0.8947
coswJ&C [35]	AnsSPL-coswJ&C	0.9679	0.9848	0.9372	0.9894	0.9654	0.9888	0.9533	0.977
Correlation values in WordNet ($\text {tree-like}_{\sigma }$ = 0.0269)									
Rada [71]	AnsSPL-Rada	0.9072	0.8882	0.9151	0.8855	0.9225	0.8994	0.9168	0.9038
Leacock and Chodorow [73]	AnsSPL-Leacock	0.9354	0.8882	0.9375	0.8855	0.937	0.8994	0.9345	0.9038
coswJ&C [35]	AnsSPL-coswJ&C	0.9993	0.9906	0.998	0.9916	0.9644	0.9859	0.9815	0.9807

We show the results obtained in the evaluation of the first 50, 100, 200, and 1000 random CUI pairs. All similarity measures are implemented in HESML V1R5 [63]. CoswJ&C [35] sets the current state-of-the-art in the family of ontology-based semantic similarity measures based on WordNet [58]. We define the tree-like deviation ($\text {tree-like}_{\sigma }$) below as the ratio of nodes with multiple parents regarding the overall number of ontology nodes. The tree-like deviation is 0 for MeSH, whilst it is (2213/82115) for WordNet 3.0, (151916/357406) for SNOMED-CT, and (19680/44509) for GO

**Table 11:** Overall running time in seconds (s) and average speed in protein pairs per second (prot. pairs/s) obtained by four groupwise GO-based similarity measures (GO, 2020-05-02 version) implemented by HESML in the evaluation of the pairwise protein similarity between the Homo Sapiens and Canis lupus familiaris organisms

Pairwise protein comparison between two large organisms			
Measure	Type	HESML Time (s)	Avg. speed (prot. pairs/s)
SimLP [100]	Common ancestors ratio	28243	12038
SimUI [100]	Common ancestor max depth	31922	10651
SimGIC-Seco [105, 110]	IC-based	30754	11055
BMA-Lin-Seco [87, 104, 110]	IC-based	7981	42604

We used the 542193 and 120720 GO annotations for both organisms provided by the “goa_human.gaf” and “go_dog.gaf” files, respectively. Approximately 340 million protein pairs and $33.5 \times 10^9$ GO-annotation pairs are compared

## Discussion

HESML outperforms by four orders of magnitude the implementation of the Rada et al. [71] path-based measure of UMLS::Similarity in the MeSH ontology as shown in Tables 7 and 9 . However, UMLS::Similarity implementation of the Rada et al. [71] measure based on caching is roughly three times faster than the HESML real-time implementation in the large SNOMED-CT ontology, as shown in Table 6. On the other hand, HESML outperforms by six and three orders of magnitude the implementation of the Lin [87] IC-based measure of UMLS::Similarity in the SNOMED-CT and MeSH ontologies respectively, as shown in Tables 6, 7 and 9. Finally, HESML outperforms by seven and four orders of magnitude the implementation of the depth-based approximation of the Wu and Palmer [72] measure of UMLS::Similarity in the SNOMED-CT and MeSH ontologies respectively, as shown in Tables 6, 7, and 9 .

HESML outperforms by six, two, and four orders of magnitude the implementation of the Rada et al. [71] path-based measure of SML in the MeSH and GO ontologies as shown in Tables 7, 8 and 9 respectively. In addition, SML is unable to provide a practical implementation of the Rada et al. [71] measure on the large SNOMED-CT ontology, as shown in Table 6. On the other hand, HESML implementation of the Lin [87] IC-based measure is roughly 2.43 times faster than the implementation of SML based on SNOMED-CT as shown in Table 6, as well as a roughly 1.55 times faster on MeSH as shown in Tables 7 and 9 , and roughly 2.86 times faster on GO as shown in Table 8.

The conclusions detailed in the two paragraphs above positively confirms our main hypothesis H1 on the outperformance of HESML on the state-of-the-art semantic measures libraries for the biomedical domain.

Path-based measures based on the new AncSPL algorithm are six and five orders of magnitude faster than their exact implementation in large ontologies with multiple inheritance, such as SNOMED-CT and GO, as shown in Tables 6 and 8 respectively, whilst AncSPL obtains similar performance to the exact implementation on tree-like ontologies like MeSH, as shown in Tables 7 and 9 , because both implementations are identical by definition. On the other hand, the results reported in Table 10 show that the reformulation of any path-based measure using AncSPL is highly correlated both in Pearson and Spearman correlation metrics with their corresponding exact implementations. High Spearman rank correlation values guarantee that any ontology-based task using ranking selection will get similar or almost identical results when AncSPL-based measures are used. Thus, this conclusion endorses the reformulation of any path-based similarity measure using AncSPL to obtain real-time approximations of any path-based measure on large ontologies with multiple inheritance, such as SNOMED-CT, GO, or WordNet. We note that in a very well-known replication of the MC30 [137] similarity benchmark carried-out by Resnik [85, Sect. 3.2], the inter-annotator Pearson correlation was 0.8848 for 30 word pairs, whilst in the most recent building of the SimLex-999 benchmark [138, Sect. 4.1] the inter-annotator Spearman correlation was 0.67 for 999 word pairs. Thus, these two later values are currently considered as reliable upper bounds of any practical estimation method for the semantic similarity between word and concepts, or like Resnik says “This value represents an upper bound on what one should expect from a computational attempt to perform the same task” [85, Sect. 3.2]. For this reason, looking at the values reported in Table 10, we can conclude that there is a high correlation between the exact path-based measures and their AncSPL reformulations.

Finally, the significant performance gain shown in Tables 6, 7, 8 and 9, together with the high-correlation values shown in Table 10, allow to confirm positively our hypothesis H2 on the performance, scalability, and approximation quality of the new AncSPL algorithm.

Groupwise similarity measures based on GO implemented by HESML provide a high average speed in the evaluation of the pairwise protein similarity between two large organisms in a large-scale experiment, as shown in Table 11. Thus, HESML can significantly contribute to improving the performance of any application using GO-based semantic similarity measures. Likewise, HESML opens the possibility of processing large-scale GO annotated data at high computation rates, which could encourage new applications like the similarity-based search of proteins in large GO-annotated databases, among others.

The shortest-path length estimated by AncSPL is always greater or equal to the exact value, as shown in Fig. 2 by the empirical Cumulative Distribution Function (CDF) for SNOMED-CT, GO, and WordNet ontologies, respectively. The signed length error of AncSPL is 0 with a probability of 0.479, 0.581, and 0.612, on SNOMED-CT, GO, and WordNet, respectively. On the other hand, the signed length error of AncSPL is less or equal to 2 with a probability of 0.874, 0.898, and 0.8841, on the three aforementioned ontologies, respectively. Thus, the AncSPL-based reformulations of any path-based similarity measure on non-tree-like ontologies always return a less or equal value than their corresponding base measures evaluated using an exact shortest-path algorithm.

The signed length error of AncSPL decreases with the tree-like deviation $(\text {tree-like}_{\sigma })$, as shown in Fig. 2. It means that lower is the number of concepts with multiple parents, higher is the probability of obtaining an AncSPL length error equals to 0. However, looking at the correlation values reported in Table 10, we can observe that correlation values obtained by the AncSPL-based reformulations in WordNet are not significantly higher than the values obtained in SNOMED-CT and GO as would be expected, with the only exception of the IC-based weighted AncSPL-coswJ&C measure, despite WordNet is close to being a tree-like ontology ($\text {tree-like}_{\sigma }$ = 0.0269). The AncSPL-coswJ&C measure obtains the higher correlation values in all ontologies and random samples, as shown in Table 10, with the only exception of the Pearson correlation for 50 concept pairs in SNOMED-CT. We conjecture that AncSPL-coswJ&C is more immune to the AncSPL approximation error than the edge-counting measures because it is defined by the length of the IC-based weighted shortest path between concepts.

The average running time of the AncSPL algorithm is linear regarding the dimension of the ancestor-based subgraph, as predicted by Theorem 1 and shown experimentally in Fig. 3 for SNOMED-CT, GO, and WordNet ontologies, respectively. As pointed out above, the performance of AncSPL depends on the dimension of the common ancestor-based subgraph and the average number of adjacent nodes for the nodes in the common ancestor-based subgraph, and not other factors as the distance between concepts, their depth in the taxonomy, or the ontology size. Likewise, the values in the third and fourth columns of Table 12 confirm that the linear time complexity of AncSPL regarding the dimension of the ancestor-based subgraph is scaled by the factor ${\bar{E}}^2_{G_{ij}}$. Looking at the third and fourth columns of Table 12, we can see that the ratio between the running-times of GO and WordNet is 1.48, whilst the expected theoretical value is 1.46, and the ratio between SNOMED and WordNet is 5.39, whilst the expected theoretical value is 7.79. These minor differences between the theoretical and experimental values for the scaling factor of $TC_3$ can be attributed to measurement noise and the removal of non-quadratic factors of ${\bar{E}}_{G_{ij}}$ to approximate its time complexity. Likewise, we conjecture that the difference is higher for SNOMED than GO, because its scalability plot is noisier, as shown in Fig. 3.

**Table 12:** Experimental confirmation of the $k{\bar{E}}^2_{C}$ factor impacting the linear scalability of AncSPL for non-tree-like ontologies ($TC_3$) shown in Fig. 3

Ontology	${\bar{E}}_{C}$	$\widehat{k{\bar{E}}^2_{C}}$ $({\mu }s)$	${\bar{E}}^2_{C}/{\bar{E}}^2_{WN}$	$\widehat{{\bar{E}}^2_{C}/{\bar{E}}^2_{WN}}$
SNOMED-CT	72.02	1.191	7.79	5.39
GO	31.14	0.3277	1.46	1.48
WordNet (WN)	25.80	0.2210	1	1

First column shows the average number of adjacent nodes per ancestor set for each node in ontology *C*, denoted by ${\bar{E}}_{C}$. Second column shows the estimated value for the factor $k{\bar{E}}^2_{C}$ in $TC_3$ obtained by fitting the scalability plot shown in Fig. 3 to the line $t_{{\mu }s} = \alpha + (k{\bar{E}}^2_{C})N$. Then, third and fourth columns compare the theoretical and experimental expected ratios between the time complexity (slope) of two different ontologies using WordNet (WN) as baseline

*Next developments planned for HESML.* As forthcoming activities, we plan to implement further tools and functionality as follows: (1) a R-package to make the HESML functionality accessible from the R program; (2) further GO-based semantic similarity measures; (3) support of further pre-trained word embeddings models for the biomedical domain; and (4) gene clustering methods among others.

## Conclusions

We have introduced a new semantic measures library for the biomedical domain called HESML V1R5, which implements the largest set of ontology-based semantic similarity measures and IC models for the SNOMED-CT, MeSH, GO, WordNet and OBO-based ontologies, as well as a new approximated shortest-path algorithm called AncSPL which provides a real-time and highly-correlated reformulation of any path-based semantic similarity measure. Our reproducible experiments show that HESML significantly outperforms current state-of-the-art semantic measures libraries in the real-time evaluation of semantic similarity measures. Likewise, our new aforementioned AncSPL algorithm allows for the first time the real-time evaluation of any path-based semantic measures, such as the large set of measures based on AncSPL which are implemented by HESML V1R5. In addition, we show that AncSPL linearly scales regarding the dimension of the common ancestor subgraph regardless of the ontology size, and the AncSPL reformulations of path-based measures are up to six and five orders of magnitude faster than their exact implementation in SNOMED-CT and GO ontologies, respectively.

The main features of HESML V1R5 are as follows: (1) the implementation of a very large set of semantic similarity methods, IC models, biomedical ontologies, and WordNet, into a single software library; (2) a real-time performance and linear scalability as regards the ontology size; (3) an open and easily extensible architecture based on abstract Java interfaces; and finally, (4) its implementation based on a portable and first-class object-oriented programming language like Java. For this reason, HESML V1R5 is a valuable resource with a huge potential for the development of high-throughput experiments and data-intensive applications in the fields of genomics and biomedical text mining.

As forthcoming activities, we plan to develop a library of sentence similarity measures for a biomedical survey [41], and Python and R interfaces for HESML.

## Supplementary Information

**Additional file 1**: We provide the Appendix A entitled "The reproducible benchmarks of biomedical semantic measures libraries" as supplementary material in one additional file. Appendix A introduces a detailed experimental setup, which is based on a publicly available reproducibility dataset [65] provided as supplementary material to allow the exact replication of all the experiments and results reported herein, as well as providing the source code of our benchmarks.

## Data Availability

In addition to the distribution of the HESML software library detailed below, we also provide a self-contained reproducibility dataset [65], together with a detailed reproducibility protocol introduced in Appendix A (see Additional file 1) to allow the exact replication of all our experiment and results. Project name: HESML. Project home page: http://hesml.lsi.uned.es/, https://github.com/jjlastra/HESML. Community forum: hesml+subscribe@googlegroups.com, hesml+unsubscribe@googlegroups.com. Current version (this work): HESML V1R5 [63]. Operating system(s): Platform independent. Programming language: Java. Other requirements: Java 1.8. License: CC By-NC-SA-4.0. Any restrictions to use by non-academics: no restrictions for non-commercial use. For commercial use of the software, it is needed to contact the authors and/or the UNED technology transfer office.

## Footnotes

1 https://www.nlm.nih.gov/.

2 http://www.obofoundry.org.

3 http://hesml.lsi.uned.es.

4 https://docs.oracle.com/javase/7/docs/api/java/util/PriorityQueue.html.

5 https://github.com/jjlastra/HESML.

6 http://current.geneontology.org/products/pages/downloads.html.
