# Supplementary material for: HESML: a real-time semantic measures library for the biomedical domain with a reproducible survey
Source: BMC Bioinformatics. 2022 Jan 6;23:23. doi: 10.1186/s12859-021-04539-0 (PMC8734250; doi:10.1186/s12859-021-04539-0)
Supplement: Supplementary file 1 — Additional file 1: We provide the Appendix A entitled "The reproducible benchmarks of biomedical semantic measures libraries" as supplementary material in one additional file. Appendix A introduces a detailed experimental setup, which is based on a publicly available reproducibility dataset [65] provided as supplementary material to allow the exact replication of all the experiments and results reported herein, as well as providing the source code of our benchmarks. [file 12859_2021_4539_MOESM1_ESM.pdf]

# Supplementary material for "HESML: a real-time semantic measures library for the biomedical domain with a reproducible survey"

Juan J. Lastra-Díaz *[1], Alicia Lara-Clares[1], and Ana García-Serrano[1]

[1]NLP & IR Research Group, E.T.S.I. Informática
Universidad Nacional de Educación a Distancia (UNED)
C/Juan del Rosal 16, 28040 Madrid (Spain)

April 30, 2021

## A The reproducible benchmarks of biomedical semantic measures libraries

This appendix introduces a detailed reproducibility protocol based on a Docker image and our supplementary reproducibility dataset [5] detailed in table A.1 with the aim of allowing the exact replication of the experiments reported in tables 6 to 10 of this work [4]. These latter data tables introduce the results of a benchmark between the UMLS::Similarity [7], SML [3] and HESML[1] V1R5 [6] (this work) semantic measures libraries based on the comparison of their performance in the evaluation of the degree of similarity between UMLS [2] CUI pairs on the SNOMED-CT, MeSH and WordNet ontologies, and GO concept pairs on the Gene Ontology [1, 10].

Our benchmarks compares the performance of the UMLS::Similarity [7], SML [3] and HESML V1R5 semantic measure libraries in five experiments using a common software platform based on a Java console program as shown in figure A.1. UMLS::Similarity is implemented as a collection of Perl scripts which interrogate a MySQL database storing the UMLS ontologies and all pre-computed information used by the library in runtime. The setup of the UMLS::Similarity library, as well as the setup and indexing of its companion UMLS database, are tedious and complex processes which could take up to one week for their completion. However, UMLS::Similarity is the pioneer semantic measures library based on UMLS ontologies reported in the literature, being it a valuable resource which has been extensively used by the research community. For this reason, we provide a Docker image with all software readily pre-installed with the aim of avoiding the aforementioned setup drawbacks. However, our reproducibility dataset [5] provides the source code of our benchmark program called HESML_UMLS_benchmark.jar, thus any reader can inspect our source code and compile the project with NetBeans 8.2 or any higher version. Likewise, the use of a Docker image to encapsulate our benchmarks warrants the possibility of reproducing them in the long-term. We refer the readers to the paper by Merkel [8] for an introductory review of Docker.

Table A.2 shows the version of the software used to run the experiments introduced in this work. The Docker image provides all software pre-installed, which means that it is not necessary to install them to reproduce the results of this paper. However, we detail this information herein for helping any reader who desires to reproduce our experimentation environment from scratch. Figure A.2 shows the workflow to reproduce our benchmarks and experimental results by running our Docker-based reproducibility package on UBUNTU. However, our benchmarks could be reproduced in any Docker-complaint platform, such as Windows, MacOS or any Linux-based system by following a similar setup to that introduced herein.

*Corresponding author: jlastra@invi.uned.es
[1]http://hesml.lsi.uned.es
[14]https://github.com/jjlastra/HESML/tree/HESML_V1R5_paper_experiments
[14]https://github.com/jjlastra/HESML/blob/HESML_V1R5_paper_experiments/HESML_Library/ReproducibleExperiments/Post-scripts/BMC_final_benchmark_tables.R

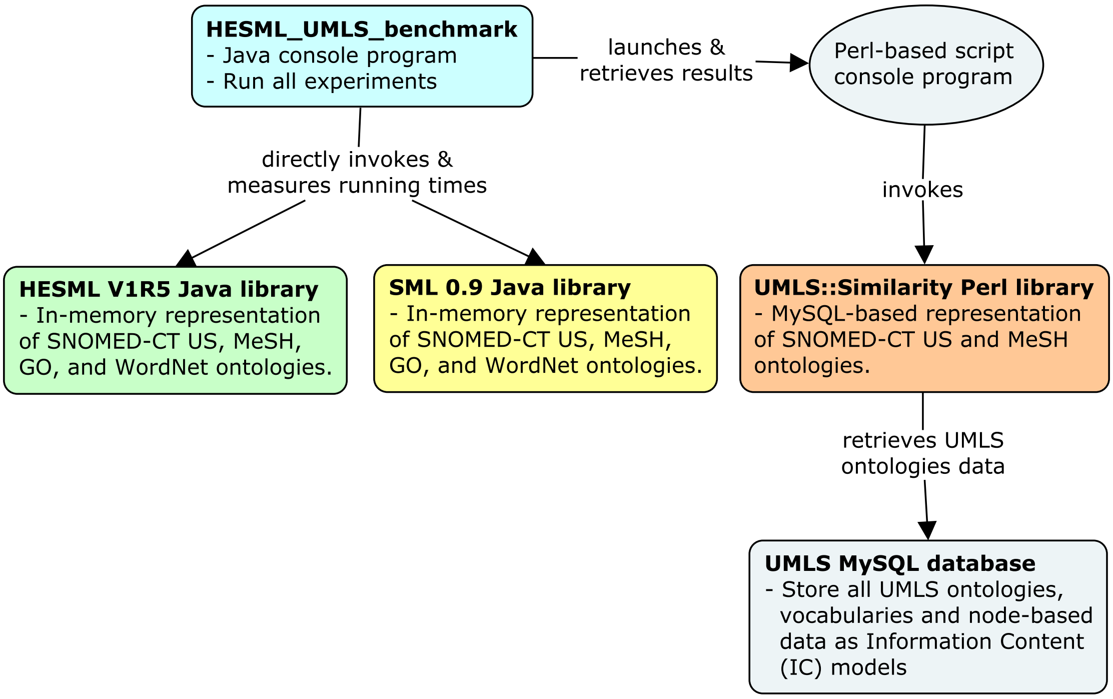


Figure A.1: Software architecture of our experimentation platform. Our benchmarks are driven by a Java console program, called *HEMSL_UMLS_benchmark*, which directly links and invokes HESML V1R5 [6] and SML 0.9 [3] Java libraries, whilst UMLS::Similarity [7] Perl library is invoked by running a Perl script console program. UMLS::Similarity stores UMLS ontologies into a MySQL database, whilst HESML and SML represent all ontologies in the main memory. The reproducible benchmarks introduced herein are based on the encapsulation of our entire experimentation platform into a Docker container based on an UBUNTU 20.04 image.

The rest of this appendix is structured as follows. Section A.1 introduced the systems requirements to reproduce our experiments. Section A.2 introduces the instructions to setup and run our experiments on any UBUNTU-based platform. Finally, section A.3 details the raw output files produced by our experiments, as well as the post-processing required to produce the collection of final data files containing all experimental results introduced herein.

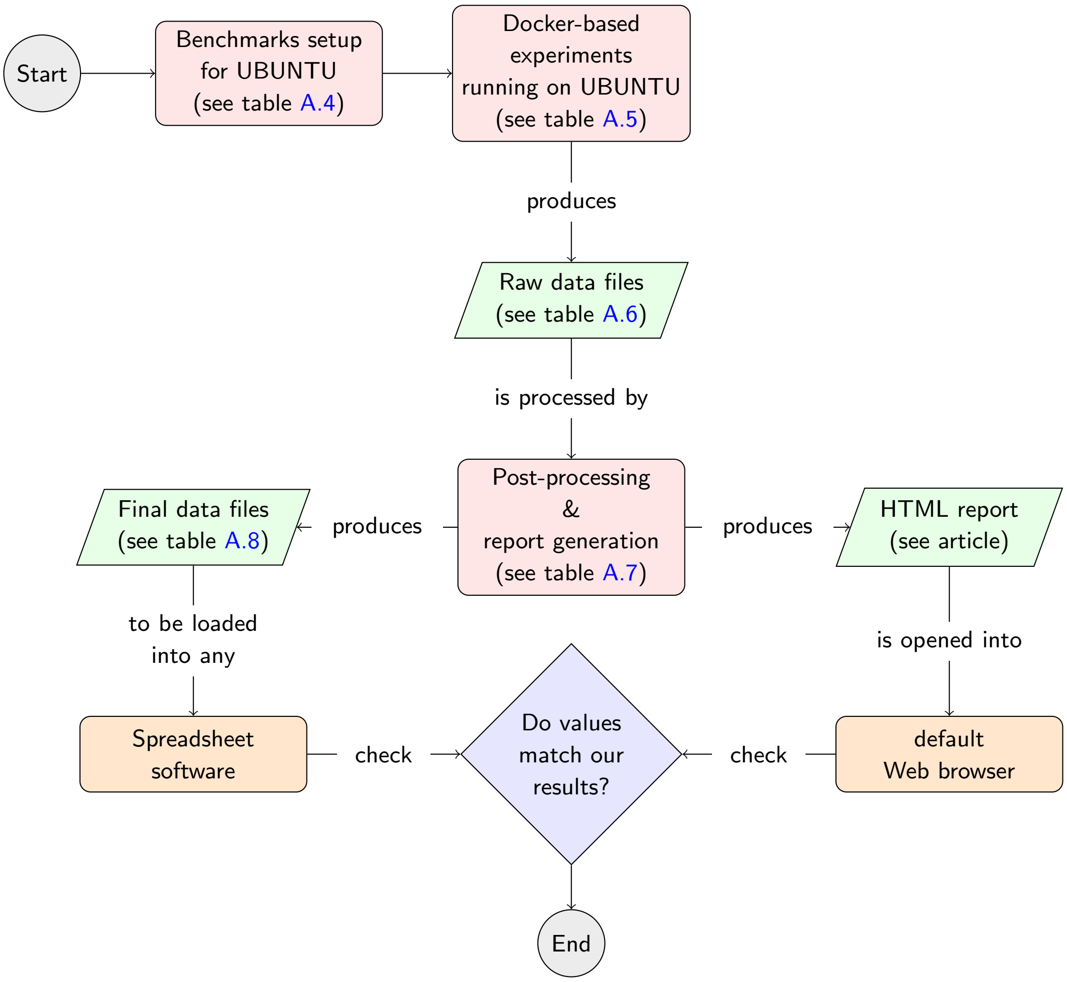


Figure A.2: Reproducibility workflow to setup and run the Docker-based reproducible experiments introduced herein on the UBUNTU operating system. The workflow detailed above produces a collection of raw and processed data files, as well as a collection of HTML pages reproducing all data tables reported herein.

| # | File | Format | Encrypted | Description |
|---|---|---|---|---|
| 1 | readme.pdf | pdf | No | Setup and reproducibility instructions (this appendix) |
| 2 | HESML_UMLS_benchmark.tar.gz | tar.gz | Yes | Java source code and binary files of our benchmark program. These files are tagged in Github as a permanent branch named "HESML_V1R5_paper_experiments" [2]. Unlike the GitHub version, this compress file contains the MedSTS dataset, which is subjected to a licensing requirement detailed below. |
| 3 | hesml-biomedical-benchmark.tar.gz | tar.gz | Yes | Docker reproducibility image based on UBUNTU |
| 4 | final_benchmark_tables.R (*) | R-script | No | R-language script for the generation of all data tables in the paper from the raw output files. |
| 5 | input_data.tar.gz | tar.gz | Yes | Input data used in our experiments on MeSH-based sentence similarity and GO-based protein similarity. This file contains the following files: (1) MedSTS_subset30_normalized.tsv (30 sentence pairs); (2) 1MPairs_NoPreprocess.txt (1 million sentence pairs), (3) MedStsFullNormalized.tsv (MedSTS dataset); (4) goa_human.gaf (GO annotations for the Homo Sapiens proteins); (5) goa_dog.gaf (GO annotations for the Canis lupus familiaris proteins). |
| 6 | rawoutput-run1.tar.gz | tar.gz | No | Raw output files used to generate all results reported in tables 6 to 11 and figures 2 and 3 of the paper. |
| 7 | rawoutput-run2.tar.gz | tar.gz | No | Raw output files from the second run of our experiments. |
| 8 | rawoutput-run3.tar.gz | tar.gz | No | Raw output files from the third run of our experiments. |

Table A.1: Detailed list of files contained in our reproducibility dataset [5]. LICENSE NOTICE: to obtain a decryptation password for the encrypted files above, you should obtain and sign a license from the National Library of Medicine (NLM) of the United States to use the UMLS Metathesaurus databases, as well as SNOMED-CT and MeSH ontologies included in our Docker image. Likewise, you should obtain and sign a Data User Agreement from the Mayo Clinic to use the MedSTS dataset by contacting the authors [11]. For this purpose, you should follow the instructions in the NLM license page, `https://uts.nlm.nih.gov//license.html`. After that, you should write to eciencia@consorciomadrono.es to obtain the password to decrypt the files. (*) In the Github project of HESML, the R script post-processing file is renamed as "BMC_final_benchmark_tables.R" [3]

| # | Software | Type | Version | Description |
|---|---|---|---|---|
| 1 | Ubuntu | Operating system | 20.04.2 LTS | |
| 2 | Docker | App deployment platform | 20.10.5 | |
| 3 | MySQL | UMLS database service | Ver 14.14<br>Distrib 5.7.30 | |
| 4 | Java | Programming Language | Openjdk 1.8.0_252 | |
| 5 | Perl | Runtime library | Ver 5.22.1 | |
| 6 | bioc-1.0.1.jar | JAR file | Ver 1.0.1 | BioC Java wrapper [4] |
| 7 | biolemmatizer-core-1.2.jar | JAR file | Ver 1.2 | Lemmatization tool [5] |
| 8 | context-2012.jar | JAR file | Ver 2012 | Metamap dependency [6] |
| 9 | json-20180813.jar | JAR file | Ver 2018 | JSON data format [7] |
| 10 | log4j-api-2.1.jar | JAR file | Ver 2.1 | Metamap dependency [8] |
| 11 | log4j-core-2.1.jar | JAR file | Ver 2.1 | Metamap dependency [9] |
| 12 | lvgdist-2020.0.jar | JAR file | Ver 2020 | Metamap dependency |
| 13 | medpostskr-1.0.jar | JAR file | Ver 1.0 | Metamap tagger |
| 14 | metamaplite-3.6.2rc5.jar | JAR file | Ver 3.6.2rc5 | Metamap library |
| 15 | mysql-connector-java-8.0.20.jar | JAR file | Ver 8.0.20 | MySQL Java wrapper [10] |
| 16 | nlp-2.4.C.jar | JAR file | Ver 2.4 | Metamap dependency [11] |
| 17 | opencsv-2.3.jar | JAR file | Ver 2.3 | CSV reader for Java [12] |
| 18 | opennlp-maxent-3.0.3.jar | JAR file | Ver 3.0.3 | Apache OpenNLP [13] |
| 19 | opennlp-tools-1.5.3.jar | JAR file | Ver 1.5.3 | Apache OpenNLP Tools [14] |
| 20 | slib-dist-0.9-all-jar.jar | JAR file | Ver 0.9 | Metamap dependency |

Table A.2: Software versions of the tools used to run the experiments introduced in this work.

## A.1 System requirements and performance evaluation

Table A.3 shows the testing platforms, as well as their the minimal requirements, in which we have successfully reproduced the experiments detailed herein, as well as their overall running times for the completion of our experiments in five different runs. Despite the size of our Docker image is 310 Gb, its deployment into a Docker container onto the host computer requires approximately 600 Gb of free disk space. Thus, the minimal hardware setup to deploy our experiments is a fresh UBUNTU computer with at least 700 Gb of disk space and 16 Gb of RAM, such as the Ubuntu-cloud computer detailed in table A.3.

**Sequential and multithreading execution modes** Due to the large execution times of some experiments in this work, HESML_UMLS_benchmark program implements a multithreading mode to parallelize the execution of the experiments that support it. Both sequential and multithreading modes maintain ratios in all library results. The sequential mode achieves better results, but increases the final accumulated run time of the experiments, as shown in Table A.3.

| Run | Test platform | Type | Mode | Operating system | Configuration | Running time |
|---|---|---|---|---|---|---|
| 1 | Ubuntu-base | Desktop | Sequential | Ubuntu 20.04 | AMD Ryzen 7 5800x (8 core), 64GB RAM, 2TB SSD disk | 375769 secs $\approx$ 4.34 days |
| 2 | Ubuntu-base | Desktop | Multithread | Ubuntu 20.04 | AMD Ryzen 7 5800x (8 core), 64GB RAM, 2TB SSD disk | 279308 secs $\approx$ 3.2 days |
| 3 | Ubuntu-cloud | Virtual | Multithread | Ubuntu 20.04 | 2 Intel Core Xeon E5 2699-v4 CPU @2.2 GHz, 16 Gb RAM, 700 Gb SSD disk | 1000603 secs $\approx$ 11.5 days |

Table A.3: Testing platforms successfully used to reproduce our experiments together with the overall running time for the completion of the reproducible experiments introduced herein. Virtual computers are cloud-based servers rented to `clouding.io`, which are based on the OpenStack virtualization platform [9].

## A.2 Setup and running instructions on UBUNTU

| Step | UBUNTU-based setup instructions for our benchmarks based on a Docker image |
|---|---|
| (1) | Create a directory to set up the experiments wherever you want and move into it<br>$ mkdir EXPS<br>$ cd EXPS |
| (2) | If Docker is not installed in your machine, instructions below install latest version of Docker CE. For further details, we refer the reader to the official Docker setup page https://docs.docker.com/install/linux/docker-ce/ubuntu/. If you copy and paste the instructions below into your command console, check the white spaces, neutral double quotes, and underline characters.<br>$ sudo apt-get -y install apt-transport-https ca-certificates<br>$ sudo apt-get -y install curl gnupg-agent software-properties-common<br>$ curl -fsSL https://download.docker.com/linux/ubuntu/gpg \| sudo apt-key add -<br>$ sudo add-apt-repository<br>"deb [arch=amd64] https://download.docker.com/linux/ubuntu $(lsb_release -cs) stable"<br>$ sudo apt-get update<br>$ sudo apt-get -y install docker-ce docker-ce-cli containerd.io<br>$ sudo docker run hello-world |
| (3) | Steps below detail how to download and decrypt our Docker image.<br>LICENSE NOTICE: to obtain a decryptation password for the last command below, you should sign and obtain a license from the National Library of Medicine (NLM) of the United States to use the UMLS Metathesaurus databases, as well as SNOMED-CT and MeSH ontologies included in our Docker image. Likewise, you should obtain and sign a Data User Agreement from the Mayo Clinic to use the MedSTS dataset by contacting the authors [11]. For this purpose, you should follow the instructions in the NLM license page, https://uts.nlm.nih.gov//license.html. After that, you should write to eciencia@consorciomadrono.es to obtain the password to decrypt the file.<br>$ sudo apt-get update<br>$ sudo apt-get install -y ccrypt<br>$ screen -S SETUP (you can detach with 'Ctrl+a, d' and reattach with 'screen -r SETUP' )<br>- The size of the encrypted file below is 66 Gb, thus the download could take several minutes.<br>$ wget https://doi.org/10.21950/hesml-biomedical-benchmark.tar.gz.cpt<br>$ ccrypt -dv hesml-biomedical-benchmark.tar.gz.cpt (it could take up to 20 minutes) |
| (4) | Step below details how to set up our Docker container to run our benchmarks. Note that there are two dash symbols before "input" in the command below.<br>$ sudo docker load –input hesml-biomedical-benchmark.tar.gz (it could takes until 1 hour) |
| (5) | Check the creation of the Docker image corresponding to the UMLS-based benchmark. If you run the command below, you should see the loaded image ID which will be needed in the next step. The created image should use a total of 310GB of disk space.<br>$ sudo docker images |
| (6) | We create and start the Docker container to run the experiments. Note that there are two dash symbols before "name" in the command below. The "IMAGE ID" value should be copied from the command (5).<br>$ sudo docker run –name=UMLS-REPRO -itd [IMAGE ID] /bin/bash<br>$ sudo docker ps -a (you should see the container UMLS-REPRO) |
| (7) | We setup MySQL within the Docker container. Step below could takes a few minutes.<br>$ sudo docker exec -it UMLS-REPRO chown -R mysql:mysql /var/lib/mysql /var/run/mysqld |

Table A.4: Detailed setup instructions on downloading and installing our benchmarks on an UBUNTU platform.

## A.3 Raw output files and post-processing

The running of our experiments as detailed in figure A.2 generates the collection of comma-separated files (*.csv) listed in table A.6, whose values are separated by a semicolon. All raw output files are saved in the same output directory in the Docker container and extracted from it by using a "docker cp" command as detailed in step 7 of table A.5.

Raw output files generated by our experiments provide different data depending on the nature of the experiment as follows: (1) running times in seconds for some evaluations of a same experiment; (2)

| Step | UBUNTU-based instructions to run our benchmarks on a Docker container |
|---|---|
| (1) | We attach to the Docker container using the screen session created above.<br>$ sudo docker attach UMLS-REPRO (pulse two times the ENTER key) |
| (2) | We are in the Docker container console, then we start MySQL server and check its status.<br>docker:$ service mysql start<br>docker:$ service mysql status (you should see "/usr/bin/mysqladmin Ver 8.42") |
| (3) | We change to the experiment directory and delete all original raw output files contained both in the output directory and a tar archive.<br>docker:$ cd /home/user/HESML_V1R5/HESML_Library/HESML_UMLS_benchmark |
| (4) | We start the experiments.<br>docker:$ java -jar -Xms8096m dist/HESML_UMLS_benchmark.jar [sequential/multithreading] |
| (5) | Experiments will take up to 30 hours. Thus, you should detach from 'screen' session by clicking Ctrl + a, d and reattach to check their completion by running the command below.<br>$ screen -r SETUP |
| (6) | Once the experiments have been completed, all raw output files are located in /home/user/HESML_V1R5/HESML_Library/ReproducibleExperiments/HESMLV1R5_paper/RawOutputFiles directory of the Docker container. First, we build a tar archive with all raw output files as detailed below:<br>docker:$ cd ../ReproducibleExperiments/HESMLV1R5_paper/RawOutputFiles<br>docker:$ tar -cvzf rawoutput.tar.gz . |
| (7) | Then, you should detach from screen console (Ctrl + a,d) in the Docker contained and execute the command below from the host console (your machine) to extract all raw output files onto any desired output directory as follows:<br>$ sudo docker cp UMLS-REPRO:/home/user/HESML_V1R5/HESML_Library/ReproducibleExperiments/ HESMLV1R5_paper/RawOutputFiles/rawoutput.tar.gz . |
| (8) | To uninstall our benchmarks, you should detach from "screen" console and follow the steps below:<br>$ sudo docker stop UMLS-REPRO<br>$ sudo docker rm UMLS-REPRO<br>$ sudo docker rmi [Image ID]<br>$ sudo docker system prune |
| (9) | Finally, you should follow the post-processing instructions detailed in section A.3 to obtain the final data tables from the raw output files retrieved in step 7 above. |

Table A.5: Detailed setup instructions on running our benchmarks on an UBUNTU platform.

a collection of similarity values corresponding to the evaluation of a collection of random CUI pairs in SNOMED-CT ontology; and (3) a collection of exact and approximated (AncSPL) shortest-path length values for a set of random SNOMED-CT concepts to carry out an statistical analysis of the AncSPL algorithm. Thus, our raw output files must be processed to compute the average running times, the Pearson and Spearman correlation values, and all statistical data shown in the tables and figures of this work. We provide a R-language script called "*final_benchmark_tables.R*" in our reproducibility dataset [5]to automate this post-processing.

In order to carry-out the aforementioned post-processing, you should set up the well-known R statistical program[15], or RStudio[16] in your workstation and follow the steps detailed in table A.7. Both statistical programs are freely available for Windows and UBUNTU. Then, you need to install the "knitr" and "readr" packages using the functionality provided for this task by R or RStudio programs. Table A.8 shows the output files which are generated from the raw output files listed in table A.6 by running our aforementioned post-processing script, as well as their corresponding data tables in the main body of this paper. In addition, our post-processing script generates a collection of HTML files which show all data tables reported herein.

[15]https://www.r-project.org/
[16]https://rstudio.com/

| In paper | Description of the experiment | File# | Raw output files |
|---|---|---|---|
| Table 6 | Similarity evaluation of random CUI pairs based on SNOMED-CT | 1 | raw_output_Rada_SNOMEDCT_US.csv |
| | | 2 | raw_output_AncSPLRada_SNOMEDCT_US.csv |
| | | 3 | raw_output_Lin_SNOMEDCT_US.csv |
| | | 4 | raw_output_WuPalmerFast_SNOMEDCT_US.csv |
| Table 7 | Similarity evaluation of random CUI pairs based on MeSH | 5 | raw_output_Rada_MeSH.csv |
| | | 6 | raw_output_AncSPLRada_MeSH.csv |
| | | 7 | raw_output_Lin_MeSH.csv |
| | | 8 | raw_output_WuPalmerFast_MeSH.csv |
| Table 8 | Similarity evaluation of random GO concept pairs | 9 | raw_output_Rada_GO.csv |
| | | 10 | raw_output_AncSPLRada_GO.csv |
| | | 11 | raw_output_Lin_GO.csv |
| Table 9 | Evaluation of sentence similarity based on MeSH, MedSTS dataset, and BioC corpus | 12 | raw_output_Rada_MedSTS.csv |
| | | 13 | raw_output_AncSPLRada_MedSTS.csv |
| | | 14 | raw_output_Lin_MedSTS.csv |
| | | 15 | raw_output_WuPalmerFast_MedSTS.csv |
| Table 10 | Pearson and Spearman correlation values between AncSPL-based reformulations and their original measures based on SNOMED-CT | 16 | raw_output_AncSPLRada_exp4.csv |
| | | 17 | raw_output_AncSPLLeacockChodorow_exp4.csv |
| | | 18 | raw_output_AncSPLCosineNormWeighted JiangConrath_exp4.csv |
| Table 11 | Evaluation of the GO-based protein semantic similarity between two organisms (Homo Sapiens and Canis lupulus familiaris) | 19 | raw_output_SimLP_largeGO_test.csv |
| | | 20 | raw_output_SimUI_largeGO_test.csv |
| | | 21 | raw_output_SimGIC_largeGO_test.csv |
| | | 22 | raw_output_BMA-Lin-Seco_largeGO_test.csv |
| Figure 2 | Raw output files for the AncSPL-based scalability experiment | 23 | raw_SNOMED_AnsSPL_subgraph_groups.csv |
| | | 24 | raw_GO_AnsSPL_subgraph_groups.csv |
| | | 25 | raw_WordNet_AnsSPL_subgraph_groups.csv |
| Figure 3 | Exact and AncSPL shortest-path length comparison for 1000 random SNOMED-CT concepts | 26 | raw_AnsSPL_SNOMED_statisticalData_test.csv |
| | | 27 | raw_AnsSPL_GO_statisticalData_test.csv |
| | | 28 | raw_AnsSPL_WordNet_statisticalData_test.csv |

Table A.6: Collection of raw output files generated by the execution of our benchmarks grouped by the table of the paper in which appear their corresponding results. All raw output files are automatically saved by our experiment driver program onto the directory detailed in step 6 of table A.5.

| Step | Detailed postprocessing instructions based on a R-language script |
|---|---|
| (1) | Launch R 4.0.5 program or R-Studio 1.3.959 (this program requires an installation of R). |
| (2) | Install the *EnvStats, knitr, kableExtra, xtable, and rstudioapi* R packages by selecting the menu option "*Packages→Install packages*" in R program, or "*Tools→Install packages*" in RStudio. |
| (3) | Download the *"final_benchmark_tables.R"* script file from our dataset [5].<br>$ wget `https://doi.org/10.21950/final_benchmark_tables.r` |
| (4) | Select the menu option "*File->Open script*". Then, load the R-language script file downloaded in step above. |
| (5) | Edit the *rootDir*, *inputDir* and *outputDir* variables at the beginning of the script in order to set the directory containing the raw output files onto your hard drive, as well as the directory in which the final assembled data tables reported in our paper will be saved. IMPORTANT NOTE: *inputDir* and *outputDir* variables should end with slash '/' symbol. |
| (6) | Select the menu option "*Edit→Run all*" in R program, or "*Code→Run region→Run all*" in RStudio. The final assembled data tables will be saved in the output directories defined above, as detailed in table A.8. In addition, the aforementioned R script creates and opens a collection of HTML files which show all data tables in our paper as detailed in table A.8. |

Table A.7: Detailed instructions for the post-processing of the raw output files generated by our experiments. R-language script computes all average speed values reported in tables 6-9 and 11, as well as the Pearson and Spearman correlation values reported in table 10. In addition, the aforementioned script generates a collection of HTML files reproducing all data tables reported in our paper.

| File# | Post-processing output files | HTML pages | Latex file | In paper |
|---|---|---|---|---|
| 1 | table6.csv | Table6.html | Table6.txt | table 6 |
| 2 | table7.csv | Table7.html | Table7.txt | table 7 |
| 3 | table8.csv | Table8.html | Table8.txt | table 8 |
| 4 | table9.csv | Table9.html | Table9.txt | table 9 |
| 5 | table10.csv | Table10.html | Table10.txt | table 10 |
| 6 | table11.csv | Table11.html | Table11.txt | table 11 |
| 7 | figure_ALL_stat.eps | — | — | Figure 2 |
| 8 | figure_ALL_average_scal.eps | — | — | Figure 3 |

Table A.8: Collection of processed output files generated by the execution of the "final_tables.R" script file onto the *outputDir* directory and their corresponding tables in the paper.

Finally, raw output data files and processed data files shown in tables A.6 and A.8 respectively could be loaded into any spreadsheet software to carry-out any further data analysis or confirming the reproducibility of the experiments and results reported herein.

# Acknowledgments

We are grateful of Fernando González and Juan Corrales for setting up our reproducibility dataset, Hongfang Liu and Yanshan Wang for providing us the MedSTS dataset, Sebastian Hennig for testing our reproducibility protocol. UMLS CUI codes, as well as SNOMED-CT US and MeSH ontologies were used in our experiments by courtesy of the National Library of Medicine (NLM) of the United States. This work was partially supported by the UNED predoctoral grant started in April 2019 (BICI N7, November 19th, 2018).

# References

[1] M. Ashburner, C. A. Ball, J. A. Blake, D. Botstein, H. Butler, J. Michael Cherry, A. P. Davis, K. Dolinski, S. S. Dwight, J. T. Eppig, M. A. Harris, D. P. Hill, L. Issel-Tarver, A. Kasarskis, S. Lewis, J. C. Matese, J. E. Richardson, M. Ringwald, G. M. Rubin, and G. Sherlock.
Gene Ontology: tool for the unification of biology.
*Nature genetics*, 25(1):25–29, May 2000.

[2] O. Bodenreider.

The Unified Medical Language System (UMLS): integrating biomedical terminology.
*Nucleic acids research*, 32(Database issue):D267–70, Jan. 2004.

[3] S. Harispe, S. Ranwez, S. Janaqi, and J. Montmain.
The semantic measures library and toolkit: fast computation of semantic similarity and relatedness using biomedical ontologies.
*Bioinformatics*, 30(5):740–742, Mar. 2014.

[4] J. J. Lastra-Díaz, A. Lara-Clares, and A. García-Serrano.
HESML: a real-time semantic measures library for the biomedical domain with a reproducible survey.
*Submitted for publication*, 2020.

[5] J. J. Lastra-Díaz, A. Lara-Clares, and A. García-Serrano.
Reproducibility dataset for a benchmark between UMLS-based semantic measures libraries.
e-cienciaDatos, 2020.
https://doi.org/10.21950/OTDA4Z.

[6] J. J. Lastra-Díaz, A. Lara-Clares, and A. García-Serrano.
HESML V1R5 Java software library of ontology-based semantic similarity measures and information content models.
e-cienciaDatos, v2, 2020.
https://doi.org/10.21950/1RRAWJ.

[7] B. T. McInnes, T. Pedersen, and S. V. S. Pakhomov.
UMLS-Interface and UMLS-Similarity : open source software for measuring paths and semantic similarity.
In *Proc. of the Annual Symposium of the American Medical Informatics Association*, volume 2009, pages 431–435, San Francisco, CA, Nov. 2009. ncbi.nlm.nih.gov.

[8] D. Merkel.
Docker: Lightweight Linux Containers for Consistent Development and Deployment.
*Linux Journal*, 2014(239):Article No. 2, Mar. 2014.

[9] O. Sefraoui, M. Aissaoui, and M. Eleuldj.
OpenStack: toward an open-source solution for cloud computing.
*International Journal of Computer Applications in Technology*, 55(3):38–42, 2012.

[10] The Gene Ontology Consortium.
The Gene Ontology Resource: 20 years and still GOing strong.
*Nucleic acids research*, 47(D1):D330–D338, Jan. 2019.

[11] Y. Wang, N. Afzal, S. Fu, L. Wang, F. Shen, M. Rastegar-Mojarad, and H. Liu.
MedSTS: a resource for clinical semantic textual similarity.
*Language Resources and Evaluation*, pages 1–16, Oct. 2018.
